# Immune cell subsets in autoimmune polyendocrine syndrome type I

**DOI:** 10.1038/s41598-025-12634-y

**Published:** 2025-08-04

**Authors:** Shahinul Islam, Bergithe E. Oftedal, Miriam Gjerdevik, Lars Breivik, Ellen C. Røyrvik, Kari Lima, Anders P. Jørgensen, Ifunanya Nwakwuo, Jørn Skavland, Eystein S. Husebye, Anette S. B. Wolff

**Affiliations:** 1 Department of Medicine, Haukeland University hospital, Bergen, Norway; 2 Department of Clinical Science, University of Bergen, Bergen, Norway; 3 Department of Computer science, Electrical engineering and Mathematical sciences, Western Norway University of Applied Sciences, Bergen, Norway; 4 Centre for Fertility and Health, The Norwegian Institute of Public Health, Oslo, Norway; 5 Department of Genetics and Bioinformatics, The Norwegian Institute of Public Health, Oslo, Norway; 6 Department of Paediatric Medicine, Oslo University Hospital, Oslo, Norway; 7 Department of Endocrinology, Akershus University Hospital, Lørenskog, Norway; 8 Department of Endocrinology, Oslo University Hospital, Oslo, Norway; 9 Faculty of Clinical Medicine, University of Oslo, Oslo, Norway; 10 Health Research Sogn og Fjordane, Førde Hospital Trust, Førde, Norway

**Keywords:** Mass cytometry, Autoimmune polyendocrine syndrome type I, APS-I, Immune phenotyping, Immune subsets, High dimensional, Proteomic, Cell biology, Immunology, Endocrinology

## Abstract

Autoimmune Polyendocrine Syndrome Type 1 (APS-I) results from mutations in the *Autoimmune Regulator* (*AIRE)* gene and serves as a valuable model to understand autoimmunity and immune deficiency. Various studies have produced differing results on how AIRE dysfunction affects the balance of immune cell subsets in blood in humans. We conducted high-resolution whole blood immune cell subset analysis using a 36-panel mass cytometry assay in Norwegian APS-I patients (*N* = 18) and compared with age and sex-matched healthy subjects (*N* = 19). Additionally, we provide a comprehensive summary of findings from 28 other studies examining blood immune subset distributions in APS-I patients. Norwegian APS-I patients had significantly reduced numbers of B cells, which was driven by lower levels of the naïve and transitional B cell compartments. We further observed trends indicating reduced frequencies of NK cells, as well as deviations in CD16-expressing cells in several subsets in APS-I patients. Contrary to previous reports, our study did not find differences in regulatory T cell levels between APS-I patients and healthy controls. Several studies in our summary consistently reported lower frequencies of resting naïve B cells in APS-I patients and higher proportions of activated memory B cells in APS-I patients. The composition of other immune cell subsets was more diverse between studies, probably reflecting contributions by other factors like medications, ethnicity, disease complexity, insufficient sample sizes, time of sampling, ages, unique immune profiles and health conditions of the patients and controls. Future research should prioritize investigating antigen-specific responses in blood and tissues to further illuminate this complex area.

**Supplementary Information:**

The online version contains supplementary material available at 10.1038/s41598-025-12634-y.

## Introduction

Autoimmune disorders encompass a spectrum of over a hundred diseases characterized by the immune system erroneously attacking the body’s own cells, leading to tissue damage and a significant loss of function. These disorders typically arise from unfavourable genetic risk variants combined with unknown environmental triggers. Autoimmune polyendocrine syndrome type 1 (APS-I) is a rare monogenic autoimmune condition caused by mutations in the *Autoimmune Regulator (AIRE)* gene^1,2^, although other genetic determinants may influence the phenotype and severity. The AIRE protein acts as a transcriptional activator in the thymus for ectopically expressed genes, facilitating central negative selection, and is crucial for T cell tolerance^3,4^. Without this control step, autoreactive T cells fail to be deleted in the thymus and are released to the blood stream and tissues, with the potential to trigger a vast variety of organ-specific autoimmune diseases.

APS-I patients often present with multiple autoimmune manifestations and problems to clear opportunistic *Candida albicans* infections^4^. Traditionally, diagnosis of APS-I requires the presence of two out of the three components: autoimmune primary adrenal insufficiency (Addison’s disease), hypoparathyroidism, and chronic mucocutaneous candidiasis. However, patients often have multiple other endocrine and ectodermal manifestations, and the diagnosis is commonly delayed^5–8^. Screening for the presence of autoantibodies against type 1 interferons and Th17-effector cytokines, present in almost all APS-I patients, are sensitive screening tools^9–12^.

Despite its rarity, APS-I serves as a valuable model for studying autoimmunity and immune deficiency, representing a clear case of monogenic negative selection failure. Previous studies on immune cell subsets in APS-I patients have often been limited by restricted markers, and specific foci, resulting in widely varying outcomes and a lack of consensus on the major impacts of AIRE deficiency on immune cells. To enhance our understanding of immune subset distribution in these patients, we conducted a comprehensive whole blood immune profiling using a 36-panel mass cytometry approach including a substantial number of APS-I patients from a geographically confined area. By leveraging the advanced capabilities of mass cytometry, we gain unprecedented analytical power to investigate immune cell compositions within this patient group. Additionally, we summarize observed data on blood immune cells from human APS-I patients across 29 studies, including 19 studies with more than 10 APS-I subjects.

## Methods

### Patients and healthy controls

Eighteen APS-I patients (eight (44%) females, ten (56%) males; age range 20–69 years, mean 45 years) were recruited from the Norwegian Registry for Organ Specific Autoimmune Diseases (ROAS) (Table S1). All the included patients fulfilled the criteria for clinical diagnosis of APS-I, had confirmed pathogenic mutations in the *AIRE* gene and signed informed written consent for participation. All methods were performed in accordance with the relevant guidelines and regulations. All patients have been reported previously^5^. None of the included subjects were on immune modulation drugs at the time of sampling, except to restore the cortisol level for patients with Addison’s disease.

Fresh whole blood from healthy persons, anonymized in this study, was collected from 19 healthy control subjects from a similar age and sex distribution (nine (47%) females, ten (53%) males; age range 20–69 years, mean 50 years). These were obtained from Haukeland University Hospital blood bank, Bergen, Norway and all had volunteered to participate in research projects.

### Blood and immune cell preparations

Immediately after collection via venepuncture, fresh whole blood samples were stabilized using Cytodelic blood stabilizer, adhering to the manufacturer’s protocol (Cytodelics AB, Stockholm, #hC002-1000). Subsequently, these samples were cryopreserved at − 80 °C. On the analysis day, samples were thawed and processed following Cytodelics AB’s protocol with the “Whole Blood Processing Kit” (#hC002-1000), and then cryopreserved again using Cryo#20 (Cytodelics AB, #hC002-1000). This was applied for 12 APS-I patients (Batch 1–4, dataset 1) and corresponding healthy controls.

A modified protocol was employed for a subset of the APS-I samples (six out of 18 (Batch 5–6, dataset 2) and six healthy controls. This involved the depletion of granulocytes from the cell suspension prior to cryopreservation and mass cytometry analysis. This separation was achieved using magnetic-activated cell sorting with CD66abce MACS microbeads (Miltenyi Biotec, #130-092-393), in accordance with the manufacturer’s guidelines. Post-separation, the cells were cryopreserved in Cryo#20 (Cytodelics AB, #hC002-1000) and subsequently processed identically to the other samples (dataset 1) for downstream analyses. A flow chart for this study is shown in Fig. S1.

### Sample barcoding, antibody staining and mass cytometry experiment

The mass cytometry panel utilized 36 antibodies, predominantly pre-conjugated with metals sourced from Standard BioTools Inc., as detailed in Table S2. Exceptions included CD5, CD14, CD20, and CD66b, which were conjugated in-house using Standard Biotools’ Antibody Labelling Kits (#201112A, #201166B, #201116A, and #201141A), following the manufacturer’s guidelines. All antibodies underwent meticulous titration before their application in the 36-panel array.

On the day of analysis, cell samples—approximately 1 million agranulocytes (dataset 2) or 2-2.5 million total white blood cells (dataset 1)—were thawed and barcoded in accordance with the Cell-ID 20-Plex Pd Barcoding Kit User Guide (Standard Biotools, #201060). These were then organized into six batches, each containing an equal mix of APS-I patient and healthy control samples, and an internal control. To prevent non-specific binding, cells were pre-treated with 1 µL of Fc-block solution (BD) and 10U of heparin (Sigma, cat. H3393). The cells were then incubated with a 36-antibody cocktail at room temperature for 30 min. Following this, cells were washed and an intercalation solution—comprising 66.5% fresh 16% paraformaldehyde (PFA) (Thermo Scientific, cat. J19943.K2), 9.5% 10X Intracellular Staining Perm Wash Buffer (BioLegend, cat. 421002), and 125nM Iridium Intercalator (Standard Biotools, cat. 201192B)—was added and left to incubate at 4℃ overnight. After a final wash, the cells were preserved in CAS plus (Standard Biotools, #201244) until acquired using the CyTOF XT instrument (Standard Biotools).

### Data processing: normalization, de-barcoding and batch-correction

The raw CyTOF XT FCS files underwent bulk normalization using the CyTOF software, with an EQ six-element calibration bead (Eq. 6 beads) based passport facilitating the normalization process. The randomization was set to align with the uniform negative distribution (UND) in linear value, ensuring compatibility with the later used softwares. The FCS files were pre-processed by exporting them into R programming using the CATALYST package. The Premessa R package was used to align individual FCS files, correct channel names, eliminate background channels, for concatenation, and for debarcoding to obtain sample-wise individual preprocessed .fcs files. Each batch was debarcoded separately with the minimum separation threshold between 0 and 1, and the barcode channels’ intensity was rescaled following filtration. The maximum Mahalanobis distance allowed between a single cell event and the centroids was set at 30.

For data cleanup, we adhered to a standard protocol in Flowjo 10.10.0 (© Becton Dickinson & Company), focusing on event length, the 140-bead channel, and Ir191/Ir193 against time, along with Gaussian parameters (Center, Width, Offset, and Residual). The Iridium intercalator was used to label intact cells.

Upon examination, notable discrepancies were observed in the control sample for different batches. We also noted significant differences, probably due to methodological issues, between the two datasets. This inconsistency necessitated the implementation of two main datasets, and batch correction within the datasets prior to further analysis. The CD45 + CD66b- cells from all individual FCS files in each batch were imported into R/RStudio for batch correction using Cydar. The ncdfFlow and flowCore packages were used to support this process. All the markers were arcsinh transformed with a scale argument/cofactor of five before batch correction. Each of the batches was corrected separately based on the included internal control in each batch using quantile correction. The quantile-corrected data was subsequently used for downstream analysis.

Marker intensities were analysed using individual tSNE plots as quality check for each marker (flowSOM).

Figure S2 illustrates how batches 1–4 were batch corrected for dataset 1 for the lineage markers CD45, CD66b, CD3, CD4, CD8 and CD14. A similar approach was performed for the other markers and for dataset 2 (not shown).

### Gating strategy and visualization of data

FCS files for all CD45 + CD66b- cells (agranulocytes) and the immune subsets CD3- CD19 + CD56- CD14- B cells, CD3 + CD19- CD56- CD14- T cells, CD3- CD19- CD56 + CD14- natural killer (NK) cells and CD3- CD19- CD56- CD14 + monocytes from dataset 1 were exported from FlowJo 10.10.0 to R Studio. SingleCellExperiment (sce)-files were made, including information on sample name, condition, type of markers (“type” or state”), marker name and batch-ID, with the help from “Catalyst” and “flowCore”^13^. A flowSOM clustering algorithm was applied to each dataset, utilizing “type” markers (lineage markers for immune cell subpopulations) as input. The resulting meta-clusters, comprising eight individual clusters, provided valuable assistance in annotating visualizations generated through downstream multidimensional reduction analysis^13^. These meta-clusters will be called «clusters».

Multidimensional unsupervised clustering and UMAP representation were initially performed on 20,000 randomly selected cells from the CD45 + CD66b- cell populations of each individual in the APS-I patient and healthy control (HC) cohorts. Using the “Catalyst,” “flowcytoscript,” “Scater,” and “diffCyt” packages, additional histograms, bar charts, and frequency tables were generated. A multi-dimensional scaling (MDS) plot on median marker expression by sample (MDS on sample-level pseudobulks) was generated with the «pbMDSA» function in Catalyst to visualize similarities and differences between clusters and/or samples in an unsupervised manner. The same analytical approach was applied to all cells from the following subsets: B cells, T cells, NK cells and monocytes, with all markers incorporated into the UMAP clustering process.

Manual gating was performed in Flowjo 10.10.0 (Table S3 and Fig. S3 for gating-strategy).

### Statistical analysis

The “diffcyt” package was applied to perform analyses of differential abundance of clusters (“EdgeR”) and differential states within clusters (“limma”) for the unsupervised analysis. FDR-values below 0.05 were considered statistically significant.

To assess differences between the APS-I patients and healthy controls in manually gated immune cell compartments, we applied median regression analysis with adjustment for batch effects using the function bsqreg with 10000 bootstrap replicates (StataNow 18 SE). Due to small sample sizes, we also performed exact Mann-Whitney U tests separately on dataset 1 and 2 (Prism GraphPad v. 10). As sensitivity analysis, we employed the Welch t-test on each dataset (Prism GraphPad v.10). We then calculated the weighted mean of the two subsets, i.e. the weighted mean of differences in mean, using the inverse of their variances as weights. Subsequently, p-values and 95% confidence intervals (CI) were constructed using the t-distribution in which the effective degrees of freedom were estimated from the Welch-Satterthwaite approximation. We accounted for multiple comparisons in the main immune cell populations, using a Bonferroni-corrected threshold (i.e. *p* < 0.0056). We did not, however, use a strict multiple testing threshold within subgroup analyses which were largely underpowered, but interpreted the results carefully. Results were visualized in a forest-plot using Prism GraphPad v. 10. For software sources and R packages for analysis, see software reference list (after reference list in the Supplementary file).

### Immune subset profiling in APS-I patients: comparisons between this study and other studies

We conducted a Pubmed search using the terms “APECED”, “APS-I”, “autoimmune polyendocrine syndrome type I,” immune cells, T cells, B cells, NK cells, monocytes, and/or dendritic cells. Potential candidate studies were identified by reviewing their abstracts and methods to determine whether they included results from more than two patients. Additionally, we examined the reference lists of the selected studies to identify further relevant publications.

Through this process, we identified 28 studies in which the authors had performed transcriptomic, immunoglobulin-class serological, and/or immune cell analyses based on protein marker expression in APS-I patients. Of these, 18 studies included more than 10 patients. Adding the present study brings the total to 19 studies with at least 10 included cases. Notably, some of the patients in this study overlapped with those reported in the following studies: Wolff et al.^14^, Oftedal et al.^15^, Berger et al.^16^, Sjøgren et al.^17^ and Kucuka et al.^18^.

## Results

### Analysis of the major immune subsets in blood: revealing disturbances of B cells in APS-I patients

We extracted approximately 1.6 million CD45- CD66b- cells from the 12 APS-I patients in dataset 1, and the same amount from the 13 healthy subjects (Fig. S6A). For subsequent analysis of dataset 1 at this level, 20,000 cells per individual were randomly selected and pooled (downsampling) to examine the major immune cell populations. These were identified through unsupervised clustering of lineage markers, including CD3 + T cells, CD4 + T helper cells (Th), CD8 + cytotoxic T cells (Tc), γδ T cells with TCR γδ, CD19 + B cells, CD56 + NK cells, CD56 + CD3 + NKT-like cells, CD14 + monocytes, CD11c + dendritic cells (DCs), and CD123 + plasmacytoid DCs (pDCs).

**Fig. 1:**
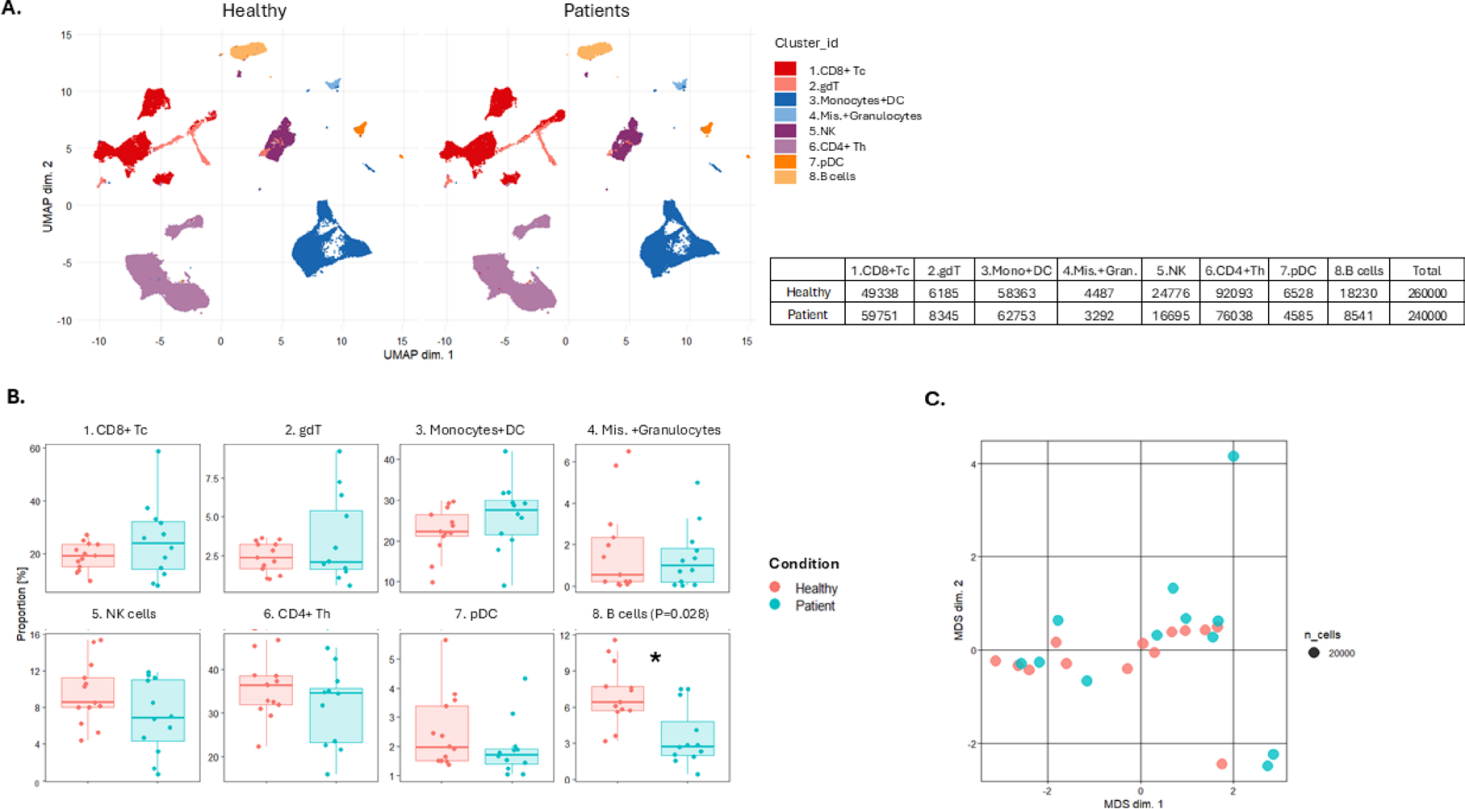
Unsupervised analysis of human whole blood-derived mass cytometry (CyTOF) normalized data on autoimmune polyendocrine syndrome type I (APS-I) patients and healthy controls (HC) for dataset 1. Downsampled CD45 + CD66b- cells. (**A**) UMAP plots including eight clusters for APS-I patients and healthy controls. The clusters are manually annotated based on the marker heatmap in Fig. S6. (**B**) Boxplot on cell frequency in each cluster for APS-I patients and healthy controls. (**C**) MDS-plot for CD45 + CD66b- cells with colours representing the two cohorts of interest. Statistical tests were performed by the EdgeR and limma R packages. Tc cytotoxic T cells, Th T helper cells, gdT gammadelta T cells, DC dendritic cells, pDC plasmacytoid DC, NK natural killer cells, Mis Miscellaneous.

Marker staining was assessed and found adequate (Fig. S4). Based on staining data from patients and healthy controls, UMAP-plots were generated, with clusters manually annotated (Fig. 1A,B, Fig. S6). Clusters 1–8 each contained more than 3000 cells for both patients and controls, providing robust clusters (Table S4). CD8 + Tc cells were slightly elevated in patients compared to healthy controls, while NK cells and pDCs were modestly reduced. However, significant differences were observed only in Cluster 8, corresponding to B cells (*p* = 0.028). Despite this, the MDS plot revealed no overall differences between the groups (Fig. 1C).

**Fig. 2:**
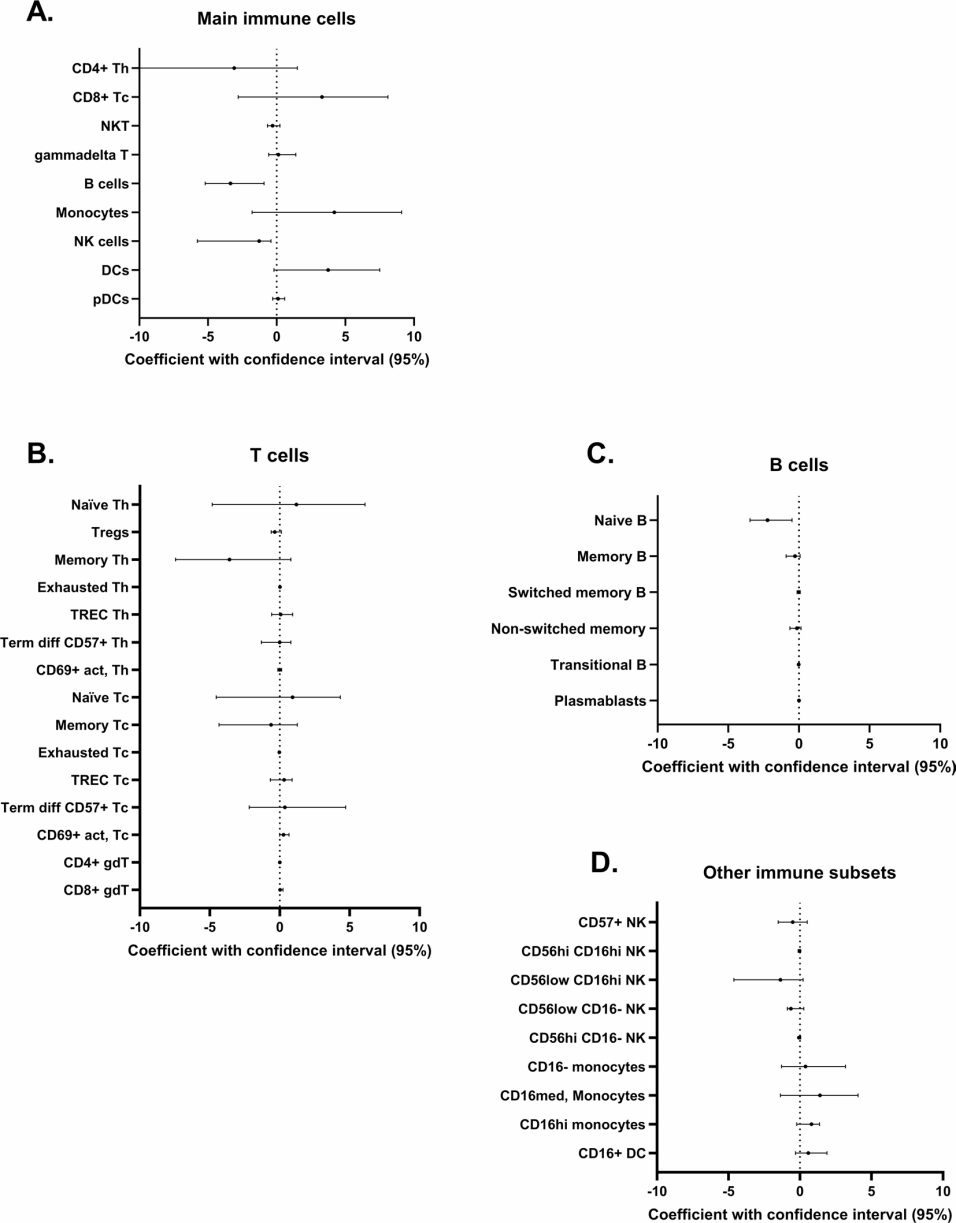
Forest plots of the observed coefficients from the median regression analyses including both dataset 1 and 2 with 95% percentile bootstrap confidence interval. Each result is given relative to healthy controls, which should be interpreted so that a positive estimate means higher in APS-I patients and a negative estimate means lower in patients. (**A**) Main immune cells. (**B**) T cell subsets. (**C**) B cell subsets. (**D**) Other immune subsets. The Figure was made by GraphPad Prism v. 10. B cells are significantly different after Bonferroni correction. Detailed statistical data can be found in Table S5.

Major immune cell subsets were further defined and manually gated in FlowJo by lineage and functional markers in both datasets 1 and 2 (Fig. 2, Fig. S5). B cells were the only population that reached statistical significance in the median regression analysis (coefficient − 3.370; CI − 5.200 to − 0.920; *p* = 0.0048). The results were also supported by our supplementary statistical analyses (Fig. S5, Table S5).

NKT-cells had lower frequencies in patients only in dataset 2 (exact Mann-Whitney, *p* = 0.0043) (Table S5, Fig. S5), but this was refuted by the unchanged frequencies for this subpopulation in dataset 1 and the joint analysis using median regression.

### Differential frequencies of B cell subpopulations in APS-I patients

**Fig. 3:**
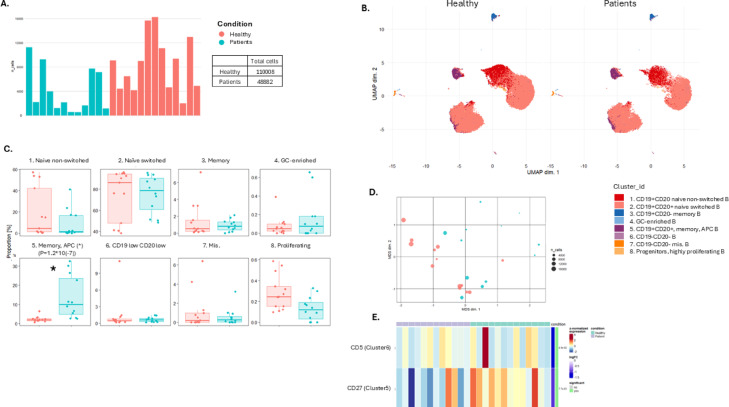
Unsupervised analysis of human whole blood-derived mass cytometry (CyTOF) normalized data on autoimmune polyendocrine syndrome type I (APS-I) patients and healthy controls (HC) for dataset 1. All B cells. (**A**) Number of B cells from each in dividual. (**B**) UMAP plots for B cell subsets including eight clusters for APS-I patients and healthy controls. The clusters are manually annotated based on the marker heatmap in Fig. S7. (**C**) Boxplot on B cell subset frequency in each cluster for APS-I patients and healthy controls. (**D**) MDS-plot for B cell subsets with colours representing the two cohorts of interest. (**E**) Statistically significantly expressed markers within clusters. Statistical tests were performed by the EdgeR and limma packages. GC germinal cernter, Mis Miscellaneous, APC antigenpresenting cells.

To look more closely into the B cell compartment, which was found overall lower in patients from our analysis on the main immune cell subgroups, we performed unsupervised analysis for dataset 1 and supervised analysis of CD3-CD19 + CD56-CD14- cells for dataset 1 and 2 (Fig. 3A–C, Fig. S5, S7, Table S4). The MDS plot for B cells visualizes differences between the groups, with patients positioned on one side and controls on the other (Fig. 3D). However, the only cluster showing a significant difference between groups was Cluster 5, corresponding to memory B cells with antigen-presenting potential (EdgeR, *p* = 1.2 × 10^−7^). Patients exhibited a higher proportion of these cells within the B cell population. Differential expression analysis at the marker level revealed higher CD5 expression in healthy controls compared to patients within Cluster 6 (limma, *p* = 4.9 × 10^−2^). Conversely, CD27, a memory marker, was more highly expressed in patients within Cluster 5 (limma, *p* = 7.7 × 10^−2^), likely contributing to the significant findings observed in this B cell cluster (Fig. 3E).

This observation was corroborated through a supervised gating strategy applied to the joint analysis of dataset 1 and dataset 2, evaluating proportions of B cell subsets of all CD45 + CD66b- cells. We revealed that the lower B cell content in APS-I is mainly driven by naïve B cells (coefficient − 2.220; CI − 3.460 to − 0.490, *p* = 0.0070). However, transitional B cells could also play a role in the diminished B cell population (coefficient − 0.020; CI −  0.048 to −  0.009, *p* = 0.043) (Fig. 2, Table S5, Fig S5, S7).

### T cell subpopulations in APS-I patients

**Fig. 4:**
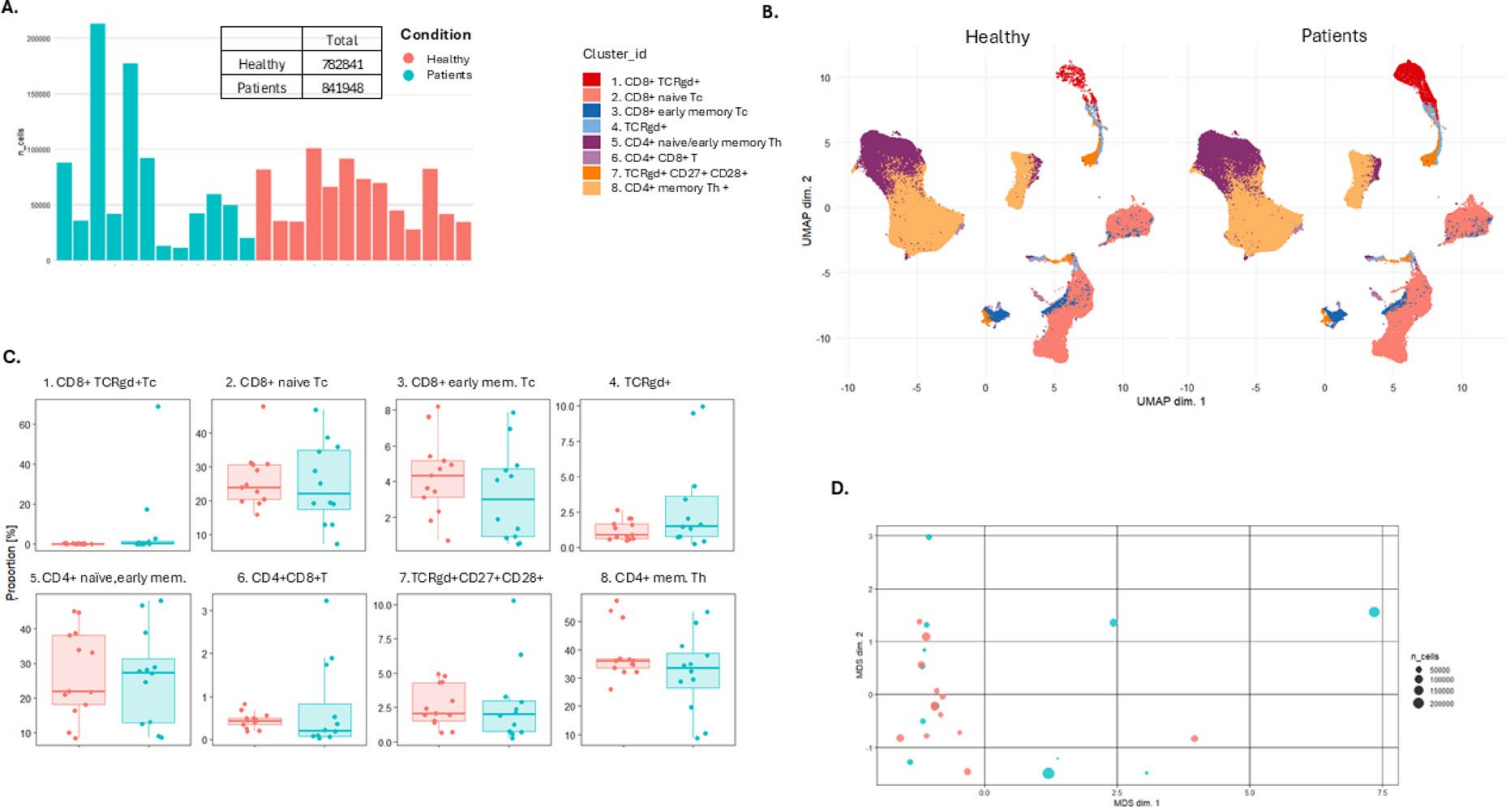
Unsupervised analysis of human whole blood-derived mass cytometry (CyTOF) normalized data on autoimmune polyendocrine syndrome type I (APS-I) patients and healthy controls (HC) for dataset 1. All T cells. (**A**) Number of T cells from each in dividual. (**B**) UMAP plots for T cell subsets including eight clusters for APS-I patients and healthy controls. The clusters are manually annotated based on the marker heatmap in Fig S7. (**C**) Boxplot on T cell subset frequency in each cluster for APS-I patients and healthy controls. (**D**) MDS-plot for T cell subsets with colours representing the two cohorts of interest. Statistical tests were performed by the EdgeR and limma packages. Th T helper cell, Tc cytotoxic T cell, mem. Memory, TCR, T cell Receptor, gdT, gammadelta T cells.

We then focused on only T cells (CD3 + CD14-CD56-CD19-) in dataset 1 and performed unsupervised cluster analysis visualized by UMAP plots (Fig. 4A–D, Fig. S7, Table S4). Most T cell clusters were similar in numbers and frequencies between the groups. However, Cluster 1, which represents CD8 + TCR γδ + cells, contained approximately 140,000 cells from patients, while fewer than 1000 cells were detected for healthy controls. This discrepancy can be attributed to two patients who were high for these otherwise rare cells, as outlined below (Table S4).

Manual subgating on T cells for dataset 1 and 2 and statistical analyses together revealed no major differences on either the CD4 + Th or the CD8 + Tc level (Fig. 2, Fig. S5, Table S5). However, the exact Mann–Whitney test for only dataset 1 revealed lower levels of PD1 + Tigit + exhausted CD8 + T cells (*p* = 0.031) and higher frequencies of CD69 + activated CD8 + T cells (*p* = 0.0031) in patients. This was not shown for dataset 2, and not for the combined analyses using median regression (Table S5). Notably, there were two samples in dataset 1 which deviated to a high extent for activated Tc cells; these “outlier” patients had 17.8% and 4.52% activated Tc cells relative to CD45 + CD66b- cells while the overall mean for healthy controls was 0.40%. The same two patients also had high levels of Tigit + Tc cells (27.90% and 5.99% while the mean for healthy controls was 2.45%) and very high frequencies of γδ T cells (16.8% and 8.39% compared to 0.70% as mean for the healthy controls), and especially high levels of CD8 + γδ T cells (16.1% and 3.42% respectively compared to the mean of healthy controls, 0.039%). These observations can probably explain the higher contribution of CD8 + γδ T cells for the UMAP plot of dataset 1 in Fig. 1 and largely affects the mean estimates of CD69 + activated CD8 + T cells in patients.

### NK cells in APS-I patients

**Fig. 5:**
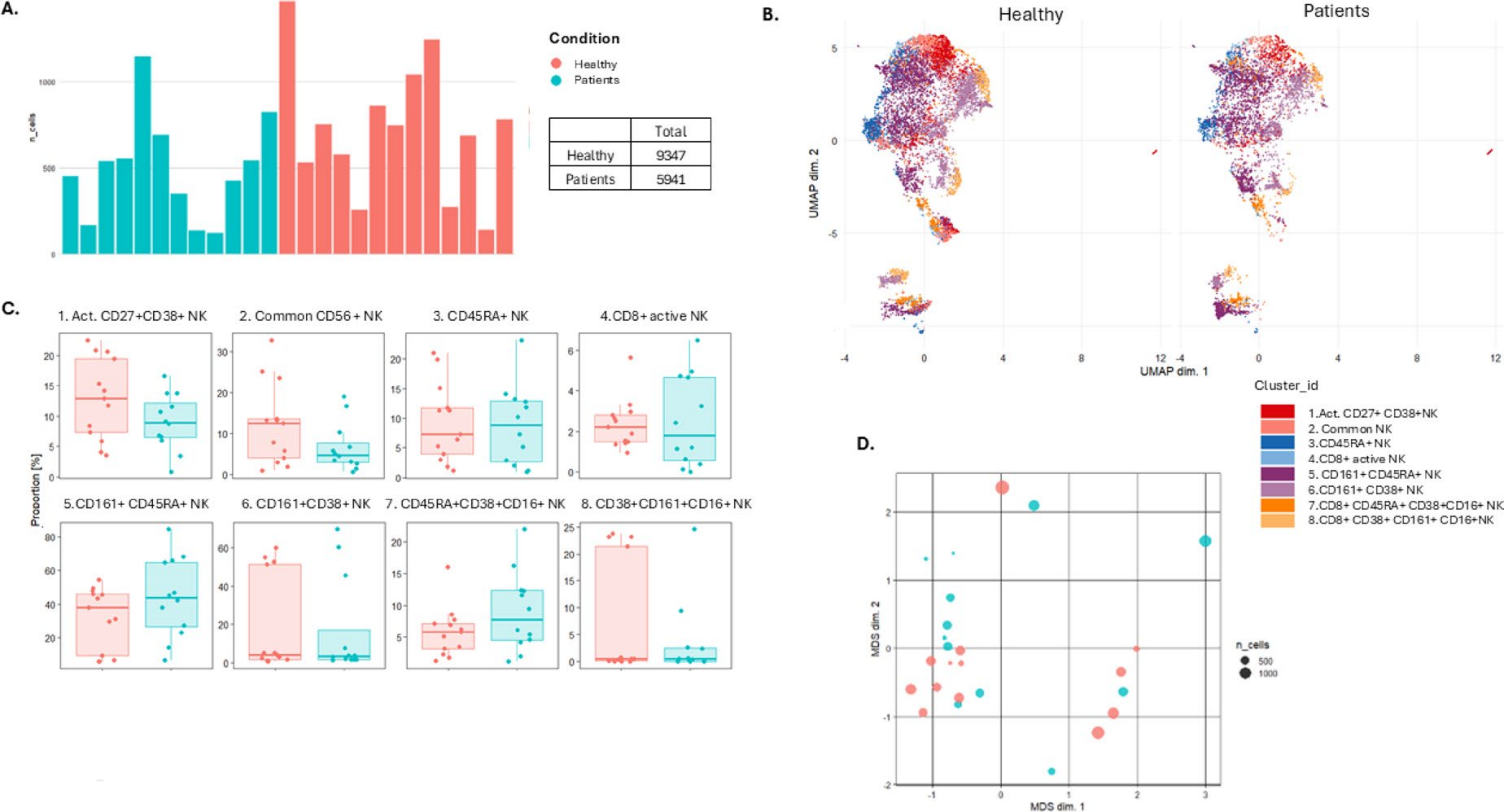
Unsupervised analysis of human whole blood-derived mass cytometry (CyTOF) normalized data on autoimmune polyendocrine syndrome type I (APS-I) patients and healthy controls (HC) for dataset 1. All natural killer (NK) cells. (**A**) Number of NK cells from each in dividual. (**B**) UMAP plots for NK subsets including eight clusters for APS-I patients and healthy controls. The clusters are manually annotated based on the marker heatmap in Fig S7. (**C**) Boxplot on NK cell subset frequency in each cluster for APS-I patients and healthy controls. (**D**) MDS-plot for NK cell subsets with colours representing the two cohorts of interest. Statistical tests were performed by the EdgeR and limma packages.

APS-I patients had a lower overall number of cells in the CD3-CD19- CD14- CD56 + NK cell compartment in dataset 1 compared to healthy donors (Fig. 5A,B, Table S4, Fig. S7), suggesting a reduced presence of NK cells in the bloodstream. However, no statistically significant differences were observed across the defined clusters (Fig. 5C,D).

NK cell subgroups were further examined through supervised manual analyses using FlowJo across both datasets. Median regression analyses revealed no significant differences in NK compartments between patients and healthy controls. However, separate Mann-Whitney analyses indicated trends towards altered frequencies of CD16 expressing NK cells, suggesting potential dysregulation with CD16 as denominator (Fig. 2, Table S5, Fig. S7). Specifically, CD56low CD16- NK were lower in patients in dataset 1 (exact Mann-Whitney, *p* = 0.0153 for dataset 1), whereas CD56low CD16 + and CD56hi CD16 + cells were reduced in patients in dataset 2 (exact Mann–Whitney, *p* = 0.0022 and *p* = 0.026, respectively) (Fig. 2 and Table S5). CD16, also known as FcγRIII, is a low affinity IgG receptor found on various immune cells, with a function in antibody-dependent cell-mediated cytotoxicity. While CD56low CD16hi NK cells are considered to have high cytotoxic capabilities, CD56hi CD16+/– cells are immunoregulatory and the CD56low CD16low NK cells are the “non-traditional” type. Thus, variations in the frequencies of CD16 expressing NK cells may have functional implications, potentially influencing immune responses in APS-I patients.

### Monocytes in APS-I patients

**Fig. 6:**
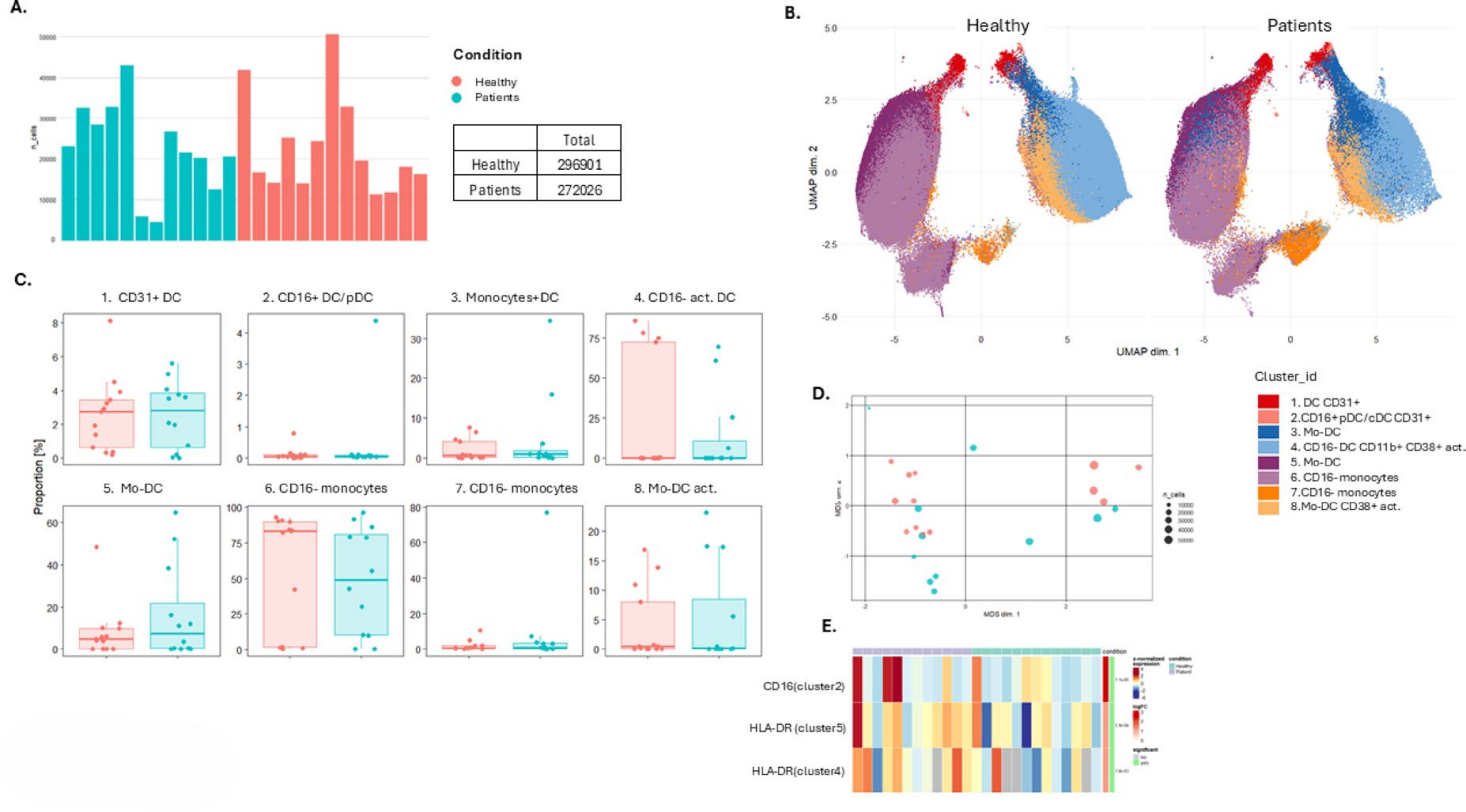
Unsupervised analysis of human whole blood-derived mass cytometry (CyTOF) normalized data on autoimmune polyendocrine syndrome type I (APS-I) patients and healthy controls (HC) for dataset 1. All Monocytes. (**A**) Number of monocytes from each in idividual. (**B**) UMAP plots for monocyte subsets including eight clusters for APS-I patients and healthy controls. The clusters are manually annotated based on the marker heatmap in Fig S7. (**C**) Boxplot on monocyte subset frequency in each cluster for APS-I patients and healthy controls. (**D**) MDS-plot for monocyte subsets with colours representing the two cohorts of interest. (**E**) Statistically significantly expressed markers within clusters. Statistical tests were performed by the EdgeR and limma packages. Th T helper cell, Tc cytotoxic T cell, mem. Memory, TCR, T cell Receptor, gdT, gammadelta T cells. Act activated, Mo monoctres, DC dendritic cells, pDC plasmacytoid DC.

The CD3- CD19- CD56- CD14 + monocyte subset was comparable between APS-I patients and healthy controls, with approximately 300,000 cells in each group (Fig. 6, Fig. S7, Table S4, S5). However, individual sample variability was substantial. No significant differential expression was observed across clusters, though a trend toward lower levels of CD16- monocytes was noted in APS-I patients (Fig. 6). Notably, CD16 expression was higher in patients within Cluster 2 (limma, *p* = 1.1 × 10⁻⁵), while HLA-DR expression was elevated in patients within Cluster 5 (Monocyte-derived DCs) (limma, *p* = 5.9 × 10^−4^) and Cluster 4 (CD16- activated DC) (limma, *p* = 1.8 × 10^−2^) (Fig. 6E).

Supervised manual analysis and gating for monocytes and DCs were conducted using FlowJo with specific attention to CD16 expression. Deviations in CD16 expression compared to healthy controls in DCs were found only in dataset 1 (exact Mann–Whitney, *p* = 0.0257). The CD16 + monocyte count was also slightly higher in APS-I patients compared to healthy controls in dataset 1, although statistical testing did not confirm significance. This was not seen for dataset 2 (Fig. 2, Fig. S5, Table S5). Furthermore, no deviation regarding HLA-DR expression as a functional marker was revealed in the supervised approach.

### Comparisons between studies/summary on the literature

**Fig. 7:**
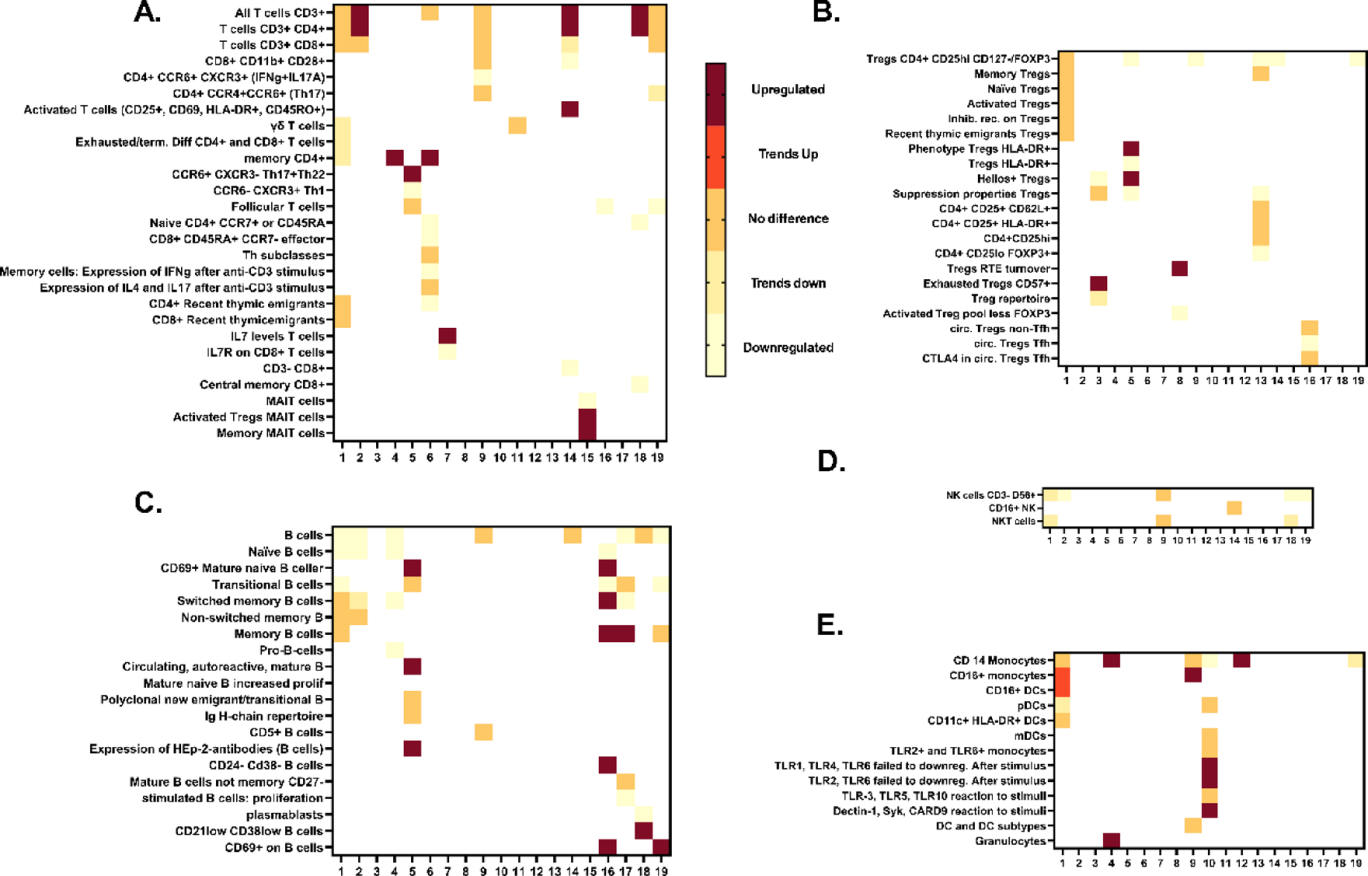
Heatmaps of the identified 19 studies with immune cell characterizations of APS-I patients, which included > 10 patients. (**A**) T cells. (**B**) Tregs. (**C**) B cells. (**D**) Natural killer (NK) cells. (**E**) Monocytes and dendritic cells (DCs). The scale goes from 0 to 4, where 0 means significantly lower, 1 means a trend towards lower, 2 means same as healthy donors, 3 means trends towards upregulation, and 4 means statistically significant upregulation. All comparisons are with healthy donors in the respective studies. The x-axis shows the number of studies, and the y-axis denotes the immune subsets. Missing values are white. A more comprehensive overview of the results in all the 29 identified studies is found in Supplementary Table 6. References for this figure. Study 1. This study. Study 2^18^. Study 3^21^. Study 4^17^. Study 5^15^. Study 6^22^. Study 7^23^. Study 8^24^. Study 9^25^. Study 10^14^. Study 11^26^. Study 12^27^. Study 13^28^. Study 14^29^. Study 15^30^. Study 16^19^. Study 17^31^. Study 18^32^. Study 19^33^.

The summary of immune cell characterization in APS-I (Fig. 7 and Table S6) highlights discrepancies between studies, likely due to limited sample sizes or distinct immune profiles within individual patients, cohorts, or ethnic groups. These inconsistencies persist even among studies examining the same patients, possibly due to insufficient statistical power (e.g. the Norwegian cohort which is included in six studies). Additionally, variations in age distribution—some studies including a higher proportion of younger versus older patients—may influence immune subset compositions. Despite these differences, the collective data convincingly demonstrates impairments in B cells and Tregs among APS-I patients. Reports on NK and NKT cells in APS-I remain inconsistent, whereas two studies have found reductions in mucosal-associated invariant T (MAIT) cells in APS-I patients^19,20^. Other immune cell subsets in the blood appear to remain unaffected in their non-activated state.

## Discussion

Immune cell phenotyping in endocrine autoimmune diseases is crucial to understand the immune dysregulation underlying these conditions. Through single cell protein marker analysis, we here demonstrate that B cells are altered in Norwegian APS-I patients at the primary immune cell level under resting conditions. Further differences from healthy controls were observed in naïve and transitional B cells, which were lower in patients. Additionally, CD16 + expression within NK cells, monocytes, and DCs appeared to show slight variation between APS-I patients and controls; however, these differences would not have reached statistical significance under multiple testing correction.We found no additional differences for the other immune subpopulations, including T cells, DCs and monocytes subsets. Both unsupervised and supervised analytical approaches were employed in this study, with most of the summarized findings derived from the supervised joint analyses of datasets 1 and 2.

While earlier studies mostly reported normal numbers and frequencies of B cells, recent research using more refined methodologies consistently highlights B cell deficiencies in APS-I patients^15,21,31,32^. Notably, naïve and transitional B cells have been identified as the most affected B cell subsets^31^, a finding corroborated by our study, though the reduction in transitional B cells was not significant. Transitional B cells likely serve as a bridge between immature bone marrow derived B cells and mature B cells in circulation and organs^34^. These cells contribute to the IL10-producing “B regulatory cells” (Bregs) pool, which has been found altered in patients with a variety of autoimmune disorders^34^. However, as no specific marker for Bregs has been conclusively identified, it remains uncertain whether APS-I patients have fewer Bregs to control autoimmunity. The observed decrease in transitional B cells suggests that B cell disturbances may originate at an immature developmental stage, potentially starting in the bone marrow. This hypothesis is supported by previous transcriptomic deconvolution analyses of blood cells from APS-I patients^15^. Notably, our recent findings indicate that differences in B cells between APS-I patients and healthy individuals are most pronounced during the early years of life, persisting until around 40 years of age^18^. However, beyond mid-life (approximately 45 years), these differences become much smaller.

The mechanism by which AIRE expression in the thymus, primarily regulating developing T cells, affects naïve B cell numbers, remains unknown and warrants further investigation. Interestingly, Sng and collaborators studied activated B cells in APS-I patients and found the frequency to be increased, possibly at the expense of Tregs^22^. Unfortunately, our study did not include some relevant B cell markers such as CD21, that could elaborate on the B cell deficiency. However, Ferre et al. reported expanded levels of CD21low CD38low B cells in APS-I patients’ blood, aligning with findings on CD21low B cells in other autoinflammatory diseases. These cells, along with chemokine receptors, influence lymphocyte trafficking and represent a distinct B cell subset^35,36^. Previous reports on APS-I, including ours, have primarily focused on non-antigen specific B cells. Future studies should compare resting and activated antigen-specific B cells to enhance our understanding of APS-I pathology.

For the T cell compartments, no major disturbances in Norwegian APS-I patients were found. In dataset 1, levels of exhausted CD8 + T cells were lower in patients, suggesting fewer “tired” T cells in the circulation compared to healthy controls. However, exhausted CD4 + T cells appeared slightly elevated in patients across both datasets. The reduction in exhausted CD8 + T cells contrasts with our and other previous findings, demonstrating that T cells from APS-I patients proliferate more rapidly and reach the terminal stages and exhaustion faster than those from healthy controls in vitro^25,37^. Furthermore, our study did not identify significant differences in frequencies of Tregs or Tregs subpopulations (not shown) in APS-I compared to the control cohort, although this cell type was slightly lower in patients in both datasets. While most prior studies examining APS-I patients have reported reduced Treg frequencies^14,25,29,37,38^, our findings may be limited by statistical power. Of notice, we recently found significant divergence in Treg frequencies among young APS-1 patients^18^. These differences tend to normalize in middle-aged patients, which is the predominant age range of our present cohort. Normal Treg-numbers for APS-1 patients have also been reported by others^31^.

NK cells are reported to be lower in number here by us (dataset 1), while we and others have shown no deviations for APS-I patients in the past^14,30,39^. Ferre and coworkers documented lower CD3- CD56 + cell counts in American APS-I patients compared to healthy donors in 2021^33^. NKT cells have been studied by others before, with findings both supporting and refuting differences from healthy controls^20,33,39^. In our analysis, dataset 2 revealed lower frequencies of NKT-like cells in APS-I patients, though these differences did not reach statistical significance when assessed via median regression analysis across both datasets. As we employed CD3 and CD56 as markers to identify the NKT-cells, this cohort may also contain conventional T cells that have gained CD56-expression upon activation. NKT cells are particularly relevant to APS-I as these cells undergo functional development in the thymus, ending up expressing the canonical Vα14-Jα18 TCRα chain linked to a limited repertoire of TCRβ chains to form their final TCR Receptor. We conclude that NK-cells and NKT-cells should be studied more in-depth to conclude their frequencies, properties, and possible contributors to disease in APS-I patients. Even though our results indicate higher frequencies of γδ T cells in APS-I patients than in controls in UMAP plots from dataset 1, the analyzed group result was probably disturbed by two patients with unique high presentation of these cell types.

CD16 is a Fc-receptor molecule that, upon recognition of antibody-coated cells, conducts signals to the NK host cell to start its killing process^40^. Hence, CD16 is a functional marker of NK cytotoxicity. When NK activation has been achieved, and cytokine production by the cell has started, the CD16 expression is decreased. A slightly lower frequency of NK cell subsets based on CD16 expression in APS-I patients may implicate that there is a reduced population of cells with killing properties. CD16 + DCSs, and partly CD16 + monocytes, were also in slightly higher frequencies in APS-I patients than controls. It is known that CD16 expression on monocytes define distinct monocytes subsets with specific functions and surface markers. An increased presence of CD16 + monocytes may tip the monocyte balance towards complement activation and phagocytosis^41,42^, consistent with proneness to autoimmunity.

Despite the relatively large patient cohort of APS-I (~ 50% of the live patients in Norway), the inclusion of more markers than any cytometry study published on these patients, and the analysis of whole blood rather than PBMCs used in other studies, several limitations must be acknowledged. First, we studied non-activated cells in blood. These findings may not accurately represent the immune status of tissues or the body as a whole. The number of subjects is further too low to stratify for age, gender and manifestations; we did however use suitable sex- and age-matched healthy controls to compare with, and run equal number of both genders and same age group in each batch. Second, the immune system is highly dynamic, and the snapshot provided by blood samples may miss transient states or the full spectrum of immune responses. Factors such as stress, infection, or medication can alter the distribution and phenotype of naive cells, potentially confounding results. Third, mass cytometry protocols present methodological constraints, particular in producing fewer events per individual compared to flow cytometry. In our unsupervised analyses, datasets were downsampled for all CD45 + CD66b- cells, and for subset analysis of B cells, T cells, NK cells and monocytes, all available markers were incorporated. We may have limited resolution compared to selecting markers specific to each subset. However, this approach was intentional, as our goal was to maintain a broad analytical scope. Lastly, as patient samples had to be handled individually during outpatient visits, technical variability may have been introduced. Direct pooling of data from the two separate datasets proved suboptimal due to variations in antibody cocktail, Cytof instruments and laboratory protocols. To address this, we applied a meta-analytic approach as a sensitivity analysis to identify immune populations that diverged between the cohorts of interest. Note that this approach relies on a normal distribution which may not hold for all cell populations. Due to small sample sizes, all results should be considered exploratory and interpreted with caution.

APS-I serves as a valuable model for endocrine autoimmunity because of its monogenic nature in regulating immune tolerance. Notably, APS-I is also a model for monogenic immune deficiency, as nearly all patients develop chronic candidiasis. Since AIRE plays a critical role in T cell tolerance, it has been speculated that APS-I may influence immune cell composition both in the bloodstream and within affected tissues. We here show that, except for the effect on B cells, the overall peripheral blood immune compartment remains largely intact in APS-I patients. We suspect more disturbances within specific T and B cell clones in activated cell states, as well as within affected tissues. In recent years, studies have examined immune cell composition in lymph nodes, mucosal tissue, skin and lung in APS-I patients^31,43–45^. Further tissue-specific investigations should be prioritized to identify target populations for therapeutic interventions.

Our study is exploratory but might help in understanding the pathogenesis of endocrine autoimmune diseases, and monitoring disease progression.

## Supplementary Information

Below is the link to the electronic supplementary material.

Supplementary Material 1

Supplementary Material 2

## Data Availability

The datasets used and/or analysed during the current study available from the corresponding author on reasonable request.
