# Supplementary material for: Immune cell subsets in autoimmune polyendocrine syndrome type I
Source: Sci Rep. 2025 Aug 4;15:28398. doi: 10.1038/s41598-025-12634-y (PMC12322070; doi:10.1038/s41598-025-12634-y)
Supplement: Supplementary file 1 — Supplementary Material 1 [file 41598_2025_12634_MOESM1_ESM.docx]

Supplementary material 1

**Immune cell subsets in autoimmune polyendocrine syndrome type I**

Shahinul Islam, Bergithe E. Oftedal, Miriam Gjerdevik, Lars Breivik, Ellen C. Røyrvik, Kari Lima, Anders P. Jørgensen, Ifunanya Nwakwuo, Jørn Skavland, Eystein S. Husebye, Anette S. B. Wolff

Table S1 **Data on the 18 included Norwegian APS-I patients**

Table S2 **Antibodies**

Table S3 **Gating strategy in table format**

Table S4 **Numbers of cells in the UMAP Clusters**

Table S5 **Descriptive data for FlowJo-manually gated immune cell subpopulations with statistics (attached excel-file: Supplementary Material 2)**

Table S6 **Overview on immune cell subsets in identified studies (N=29)**

Fig. S1 **Overview of study**

Fig. S2 **Batch correction algorithm testing (Dataset 1).**

Fig. S3 **Gating strategy for main immune cell populations**

Fig. S4 **Details of the unsupervised gating of all major immune cells from Dataset 1**

Fig. S5 **Manually gated dataset 1 and 2 analyzed separately with histograms**

Fig. S6 **Supplementary data from analysis of downsized CD45+ CD66b- cells from APS-I patients and healthy controls (Dataset 1)**

Fig. S7 **Abundance plot and marker heatmap for immune subpopulations from APS-I patients and healthy controls (Dataset 1).**

**Supplementary Table 1 (Table S1) Data on the 18 included Norwegian APS-I patients**

| **Patient ID** | **Age at sampling** | **Manifestations*** | **Sex** | **Batch¤** |
| --- | --- | --- | --- | --- |
| 1 | >60 | C, HP, A, G, E | F | 6 |
| 2 | 50-60 | C, A | M | 6 |
| 3 | 50-60 | C, HP, A, M, Al, E, K | M | 5 |
| 4 | 40-50 | C, HP, E, AT, V | F | 3 |
| 5 | 20-30 | C, HP, A, Al, AT, E | M | 2 |
| 6 | 30-40 | C, HP, A, E, Al | M | 2 |
| 7 | 30-40 | C, HP, K, M, E | M | 2 |
| 8 | 40-50 | C, HP, G, Al, E | F | 3 |
| 9 | >60 | C, HP, V, D, E, AT | M | 1 |
| 10 | 40-50 | C, A, E | F | 1 |
| 11 | 50-60 | C, HP, D, K, V, Al, E | M | 1 |
| 12 | >60 | A | F | 3 |
| 13 | 50-60 | HP, G, M, E | F | 5 |
| 14 | 50-60 | C, E | M | 5 |
| 15 | 20-30 | C, HP, A, M, E | F | 4 |
| 16 | 30-40 | C, HP, A, M, E | M | 6 |
| 17 | 30-40 | C, A, M, E | M | 4 |
| 18 | 20-30 | C, HP, A, D, Al | F | 4 |

**Manifestations*:** C: Chronic mucocutaneous candidiasis; HP: hypoparathyroidism; A: Adrenal insufficiency (Addison’s disease); G: Gonadal failure: E: Enamel failure; M: Malabsorption; Al: Alopecia; K: Keratiris; AT: autoimmune thyroid disease; V: vitiligo; D: type I diabetes. Some diagnoses, including nail pitting, Tubulointerstitial nephritis, and asplenia, are not mentioned. They are not denoted as very few patients have these manifestations; thus, they may help identify the individual patients.

**¤Batch** refers to which batch the corresponding patient sample was included in the Cytof experiments.

**Supplementary Table 2 (Table S2)**

**Antibodies**

|  | **Antibody** | **Metal** | **Clone** | **Company** | **Cat.nr** | **Present on cells** |
| --- | --- | --- | --- | --- | --- | --- |
| 1 | CD10 | 156Γδ | HI10a | Fluidigm | 3156001B | B cell subtypes |
| 2 | CD103 | 151Eu | Ber-ACT8 | Fluidigm | 3151011B | Tregs ++ |
| 3 | CD11b | 167Er | ICRF44 | Fluidigm | 3167011B | Many cell types |
| 4 | CD11c | 147Sm | Bu15 | Fluidigm | 3147008B | DCs++ |
| 5 | CD123 (IL3R) | 143Nd | 6H6 | Fluidigm | 3143014B | pDCs |
| 6 | CD127 (IL7Ra) | 149Sm | A019D5 | Fluidigm | 3149011B | not expressed on Tregs |
| 7 | CD134 (Ox40) | 150Nd | ACT35 | Fluidigm | 3150023B | Tregs++ |
| 8 | CD14 | 112Cd | MEM15 | Invitrogen, * | MA1-19223 | Monocytes |
| 9 | CD38 | 172Yb | HIT2 | Fluidigm | 3172007B | B (plasma) cells, ++ |
| 10 | CD152 (CTLA-4) | 161Dy | 14D3 | Fluidigm | 3161004B | Tregs++ (inhib.rec.) |
| 11 | CD16 | 209Bi | 3G8 | Fluidigm | 3209002B | NK, monocytes |
| 12 | CD161 | 164Dy | HP-3G10 | Fluidigm | 3164009B | NK, Th17, ++ |
| 13 | CD19 | 142^Nd^ | HIB19 | Fluidigm | 3142001B | B cells |
| 14 | CD20 | 116Cd | 2H7 | eBioScience, * | 14-0209-82 | B cells |
| 15 | CD25 (IL2R) | 169Tm | 2A3 | Fluidigm | 3169003B | Activated T cells, Tregs |
| 16 | CD27 | 158Γδ | L128 | Fluidigm | 3158010B | B/T cells |
| 17 | CD274 (PD-L1) | 159Tb | 29E.2A3 | Fluidigm | 3159029B | APCs |
| 18 | CD278 (ICOS) | 148Nd | C398.4A | Fluidigm | 3148021D | Tregs++ (inhib.rec.) |
| 19 | CD279 (PD-1) | 155Γδ | EH12.2H7 | Fluidigm | 3155009B | activated T cells |
| 20 | CD28 | 160Γδ | CD28.2 | Fluidigm | 3160003B | B/T cells |
| 21 | CD3 | 170Er | UCHT1 | Fluidigm | 3170001B | T cells |
| 22 | CD31/ PECAM1 | 144Nd | WM59 | Fluidigm | 3144023B | recent thymic emigrants |
| 23 | CD4 | 145Nd | RPA-T4 | Fluidigm | 3145001B | Th cells |
| 24 | CD45 | 89Y | HI30 | Fluidigm | 3089003B | leucocytes |
| 25 | CD45RA | 153Eu | HI100 | Fluidigm | 3153001B | naïve T cells |
| 26 | CD45RO | 165Ho | UCHL1 | Fluidigm | 3165011B | memory T cells |
| 27 | CD5 | 166Er | UCHT2 | Invitrogen, * | 14005982 | B cells |
| 28 | CD56 | 163Dy | NCAM16.2 | Fluidigm | 3163007B | NK cells, NKT cells |
| 29 | CD57 | 176Yb | HCD57 | Fluidigm | 3176019B | exhausion, term. diff. |
| 30 | CD66b | 141Pr | G10F5 | Bio-Techne, * | NBP2-80664 | Granulocytes |
| 31 | CD69 | 162Dy | FN50 | Fluidigm | 3162001B | Activation marker |
| 32 | CD8a | 168Er | SK1 | Fluidigm | 3168002B | cytotoxic T cells |
| 33 | HLA-DR | 174Yb | L243 | Fluidigm | 3174001B | Activation marker, APCs |
| 34 | Iγδ | 146Nd | IA6-2 | Fluidigm | 3146005B | B cells |
| 35 | TCRγδ | 152Sm | 11F2 | Fluidigm | 3152008B | γδ T cells |
| 36 | Tigit | 154Sm | MBSA43 | Fluidigm | 3154016B | Tregs++ (inhib.rec.) |
| * Conjugated in house; ++ Several cell types | | | |  |  |  |

**Supplementary Table 3 (Table S3) Gating strategy in table format**

| **Lineage** | **Main immune cell subsets** | **Figure** | **Level 1** | **Level 2/3/4** |
| --- | --- | --- | --- | --- |
| T | T cells | Fig. 2 | CD3+ CD14- CD19- CD56- | TCRγδ- CD4+ CD8- |
| CD4 T | T helper cells (Th) | Fig. 2 | CD3+CD14-CD19-CD56- | TCRγδ- CD4+ CD8- |
| CD8 T | Cytotoxic T cells (Tc) | Fig. 2 | CD3+CD14-CD19-CD56- | TCRγδ- CD4- CD8+ |
| CD4 T | Tregs | Fig. 2 | CD3+CD14-CD19-CD56- | TCRγδ- CD4+ CD8- CD25hi CD127- |
| γδ T | TCR γδ T cells | Fig. 2 | CD3+ CD14- CD19- CD56- | TCRγδ+ |
| NKT | NKT-like cells | Fig. 2 | CD3+CD14-CD19-CD56+ |  |
| B | B cells | Fig. 2 | CD3- CD14- CD19+ CD56- |  |
| Monocytes | Monocytes | Fig. 2 | CD3-CD14+CD19-CD56- |  |
| NK | NK cells | Fig. 2 | CD3-CD14-CD19-CD56+ |  |
| DCs | HLA-DR+ DC (Classical DC) | Fig. 2 | CD3- CD14- CD19- CD56- | CD11c+ HLA-DR+ |
| pDCs | HLA-DR+ pDCs | Fig. 2 | CD3- CD14- CD19- CD56- | CD11c- HLA-DR+ CD123+ |
| **Lineage** | **Cell Subset (Additional subsets)** | **Figure** | **Level 1** | **Level 2/3/4** |
| γδ T | CD4+ γδ T cell | Suppl. Fig. 5 | CD3+ CD14- CD19- CD56- | TCRγδ+ CD4+ |
| γδ T | CD8+ γδ T cell | Suppl. Fig. 5 | CD3+ CD14- CD19- CD56- | TCRγδ+ CD8+ |
| CD4 T | Naive Th | Suppl. Fig. 5 | CD3+ CD14- CD19- CD56- | CD4+ CD8a- TCRγδ- CD45RA+ CD45RO- |
| CD4 T | Memory Th | Suppl. Fig. 5 | CD3+ CD14- CD19- CD56- | CD4+ CD8a- TCRγδ- CD45RA- CD45RO+ |
| CD4 T | CD45RA+ CD45RO+ Th | Suppl. Fig. 5 | CD3+ CD14- CD19- CD56- | CD4+ CD8a- TCRγδ- CD45RA+ CD45RO+ |
| CD4 T | Exhausted Th | Suppl. Fig. 5 | CD3+ CD14- CD19- CD56- | CD4+ CD8a- TCRγδ- CD27- CD28+ CD45RA- PD1+ Tigit+ |
| CD4 T | Thymic recent emigrant Th | Suppl. Fig. 5 | CD3+ CD14- CD19- CD56- | CD4+ CD8a- TCRγδ- CD31+ |
| CD4 T | Term.diff. Th | Suppl. Fig. 5 | CD3+ CD14- CD19- CD56- | CD4+ CD8a- TCRγδ- CD57+ |
| CD4 T | Activated Th | Suppl. Fig. 5 | CD3+ CD14- CD19- CD56- | CD4+ CD8a- TCRγδ- CD69+ |
| CD4 T | PD1 Th (exhaustion marker) | Suppl. Fig. 5 | CD3+ CD14- CD19- CD56- | CD4+ cd8a- TCRγδ- CD279+ |
| CD4 T | Tigit+ Th (exhaustion marker) | Suppl. Fig. 5 | CD3+ CD14- CD19- CD56- | CD4+ cd8a- TCRγδ- Tigit+ |
| CD8 T | Naive Tc | Suppl. Fig. 5 | CD3+ CD14- CD19- CD56- | CD4- CD8a+ TCRγδ- CD45RA+ CD45RO- |
| CD8 T | Memory Tc | Suppl. Fig. 5 | CD3+ CD14- CD19- CD56- | CD4- CD8a+ TCRγδ- CD45RA- CD45RO+ |
| CD8 T | CD45RA+ CD45RO+ Tc | Suppl. Fig. 5 | CD3+ CD14- CD19- CD56- | CD4- CD8a+ TCRγδ- CD45RA+ CD45RO+ |
| CD8 T | Exhausted Tc | Suppl. Fig. 5 | CD3+ CD14- CD19- CD56- | CD4- CD8a+ TCRγδ- CD27- CD28+ CD45RA- PD1+ Tigit+ |
| CD8 T | Thymic recent emigrant Tc | Suppl. Fig. 5 | CD3+ CD14- CD19- CD56- | CD4- CD8a+ TCRγδ- CD31+ |
| CD8 T | Term.diff. Tc | Suppl. Fig. 5 | CD3+ CD14- CD19- CD56- | CD4- CD8a+ TCRγδ- CD57+ |
| CD8 T | Activated Tc | Suppl. Fig. 5 | CD3+ CD14- CD19- CD56- | CD4- CD8a+ TCRγδ- CD69+ |
| CD8 T | PD1 Tc (exhaustion marker) | Suppl. Fig. 5 | CD3+ CD14- CD19- CD56- | CD4- CD8a+ TCRγδ- CD279+ |
| CD8 T | Tigit+ Tc (exhaustion marker) | Suppl. Fig. 5 | CD3+ CD14- CD19- CD56- | CD4- CD8a+ TCRγδ- Tigit+ |
| B | B cells CD19+ CD20+ | Suppl. Fig. 8 | CD3- CD14- CD19+ CD56- | CD20+ |
| B | B cells Naive | Suppl. Fig. 8 | CD3- CD14- CD19+ CD56- | CD27- CD10- |
| B | B cells CD27- CD38+ | Suppl. Fig. 8 | CD3- CD14- CD19+ CD56- | CD27- CD38+ |
| B | B cells Transitional | Suppl. Fig. 8 | CD3- CD14- CD19+ CD56- | CD27- CD38+ CD10+ CD5+ Iγδ- |
| B | B cells Memory | Suppl. Fig. 8 | CD3- CD14- CD19+ CD56- | CD27+ CD10- |
| B | B cells Switched memory | Suppl. Fig. 8 | CD3- CD14- CD19+ CD56- | CD27+ CD38- Iγδ- |
| B | B cells Non-switched memory | Suppl. Fig. 8 | CD3- CD14- CD19+ CD56- | CD27+ CD38- Iγδ+ |
| B | B cells Plasmablasts | Suppl. Fig. 8 | CD3- CD14- CD19+ CD56- | CD20- CD27+ CD10- |
| NK | NK Cell (CD56hi CD16hi) | Suppl. Fig. 8 | CD3- CD14- CD19- CD56+ | CD56hi CD16+ |
| NK | NK Cell (CD56hi CD16-) | Suppl. Fig. 8 | CD3- CD14- CD19- CD56+ | CD16hi CD16- |
| NK | NK Cell (CD56low CD16+) | Suppl. Fig. 8 | CD3- CD14- CD19- CD56+ | CD56low CD16+ |
| NK | NK Cell (CD56low CD16-) | Suppl. Fig. 8 | CD3- CD14- CD19- CD56+ | CD16low CD16- |
| NK | NK Cell CD57+ (exhausted, term.diff.) | Suppl. Fig. 8 | CD3- CD14- CD19- CD56+ | CD57+ |
| Monocytes | Monocyte (CD14+ CD16hi) | Suppl. Fig. 8 | CD3- CD14+ CD19-CD56- | CD16+ |
| Monocytes | Monocyte (CD14+ CD16-) | Suppl. Fig. 8 | CD3- CD14+ CD19-CD56- | CD16- |
| Monocytes | Monocyte (CD14+ CD16med.) non-classic | Suppl. Fig. 8 | CD3- CD14+ CD19- CD56- | CD16med. |
| DCs | CD16+ Classical DCs | Suppl. Fig. 8 | CD3- CD14- CD19- CD56- | CD11c+ CD16+ |

NK: Natural killer cells; DC: Dendritic cells; p: plasmacytoid

**Supplementary Table 4 (Table S4) Numbers of cells in the UMAP meta-clusters**

The first row in each table represent meta-cluster number which corresponds to the same number in the relevant accompanied fifure (Fig. 1, 3, 4, 5 and 6)

| **A. Downsized all CD45+ CD66b- cells (20000 cells per individual)** | | | | | |  |  |  |  |
| --- | --- | --- | --- | --- | --- | --- | --- | --- | --- |
|  | 1 | 2 | 3 | 4 | 5 | 6 | 7 | 8 | Total |
| Healthy | 49338 | 6185 | 58363 | 4487 | 24776 | 92093 | 6528 | 18230 | 260000 |
| Patient | 59751 | 8345 | 62753 | 3292 | 16695 | 76038 | 4585 | 8541 | 240000 |
|  |  |  |  |  |  |  |  |  |  |
| **B. CD45+ CD66b- CD3+ CD19- CD56- CD14- T cells** | | | | |  |  |  |  |  |
|  | 1 | 2 | 3 | 4 | 5 | 6 | 7 | 8 | Total |
| Healthy | 726 | 200624 | 34239 | 8366 | 211153 | 3484 | 19366 | 304883 | 782841 |
| Patient | 139416 | 200251 | 19428 | 23587 | 186089 | 2640 | 18630 | 251907 | 841948 |
|  |  |  |  |  |  |  |  |  |  |
| **C. CD45+ CD66b- CD3- CD19+ CD56- CD14- B cells** |  |  |  |  |  |  |  |  |  |
|  | 1 | 2 | 3 | 4 | 5 | 6 | 7 | 8 | Total |
| Healthy | 29043 | 74445 | 2006 | 57 | 2339 | 1187 | 634 | 297 | 110008 |
| Patient | 5817 | 36704 | 627 | 40 | 4953 | 299 | 372 | 70 | 48882 |
|  |  |  |  |  |  |  |  |  |  |
| **D. CD45+ CD66b-CD3- CD19- CD56+ CD14- NK cells** |  |  |  |  |  |  |  |  |  |
|  | 1 | 2 | 3 | 4 | 5 | 6 | 7 | 8 | Total |
| Healthy | 1107 | 1174 | 806 | 257 | 2696 | 1909 | 611 | 787 | 9347 |
| Patient | 468 | 259 | 361 | 103 | 2491 | 1443 | 464 | 352 | 5941 |
|  |  |  |  |  |  |  |  |  |  |
| **E. CD45+ CD66b- CD3- CD19- CD56- CD14+ Monocytes** |  |  |  |  |  |  |  |  |  |
|  | 1 | 2 | 3 | 4 | 5 | 6 | 7 | 8 | Total |
| Healthy | 7472 | 436 | 9326 | 114882 | 17219 | 123186 | 4482 | 19898 | 296901 |
| Patient | 7484 | 2039 | 20144 | 52105 | 42615 | 103146 | 24263 | 20230 | 272026 |

**Supplementary Table 5 (Table S5): Descriptive data for FlowJo-manually gated immune cell subpopulations.** A quantile median regression analysis was employed to look for differences in the two datasets together between APS-I patients and healthy controls. Exact Mann-Whitney U tests were employed to investigate differences in the separate dataset 1 and 2. Statistical tests for each dataset were additionally performed using the Welch t-test in Prism Graphpad v.10. Means refer to percentages of cells with respect to CD45+CD66b- (“all cells”). The weighted mean of the two subsets, i.e. the weighted mean of differences in mean, was then computed using the inverse of their variances as weights. The mean is reported for patients with respect to healthy controls. p-values and 95% confidence intervals (CI) were constructed using the t-distribution.

*Attached Excel-file (Supplementary Material 2)*

Supplementary Table 6 (Table S6) **Identified studies for immune cell composition analysis/humoral response in APS-I patients (N=29)**

1. **Studies of immune cell compositions in APS-1 (N>10 within the study)**

| **Level** | **Immune molecule/subset** | **Score** | **Normal** | **Up** | **Down** | **Functional flaws** | **# APS-1** | **Study** | **Ref** |
| --- | --- | --- | --- | --- | --- | --- | --- | --- | --- |
| Cells/protein | All T cells CD3+ | 2 | x |  |  |  | 19 | Explorative | This study (Islam et al) |
| Cells/protein | T cells CD3+ CD4+ | 2 | x |  |  |  | 19 |  |  |
| Cells/protein | T cells CD3+ CD8+ | 2 | x |  |  |  | 19 |  |  |
| Cells/protein | γδ T cells | 2 | x |  |  |  | 19 |  |  |
| Cells/proteins | Exhausted/term. Diff CD4+ and CD8+ T cells | 3 | (x) | (x) |  |  | 19 |  |  |
| Cells/proteins | Memory CD4+ | 1 | (x) |  | (x) |  | 19 |  |  |
| Cells/protein | Tregs CD4+ CD25hi CD127- | 2 | x |  |  |  | 19 |  |  |
| Cells/protein | memory Tregs | 2 | x |  |  |  | 19 |  |  |
| Cells/protein | Naïve Tregs | 2 | x |  |  |  | 19 |  |  |
| Cells/protein | Activated Tregs | 2 | x |  |  |  | 19 |  |  |
| Cells/protein | Inhib. rec. on Tregs | 2 | x |  |  |  | 19 |  |  |
| Cells/protein | TREC Tregs | 2 | x |  |  |  | 19 |  |  |
| Cells/protein | B cells | 0 |  |  | x |  | 19 |  |  |
| Cells/protein | Naïve B cells | 0 |  |  | x |  | 19 |  |  |
| Cells/protein | Transitional B cells | 0 |  |  | x |  | 19 |  |  |
| Cells/protein | Switched memory B cells | 2 | x |  |  |  | 19 |  |  |
| Cells/protein | Memory B cells | 2 | x |  |  |  | 19 |  |  |
| Cells/protein | NK cells and CD16+ subtype | 1 | (x) |  | (x) |  | 19 |  |  |
| Cells/protein | NKT-like cells | 1 | (x) |  | (x) |  | 19 |  |  |
| Cells/protein | Monocytes | 2 | x |  |  |  | 19 |  |  |
| Cells/protein | CD16+ monocytes | 3 | (x) | (x) |  |  | 19 |  |  |
| Cells/protein | CD16+ DCs | 3 | (x) | (x) |  |  | 19 |  |  |
| Cells/protein | pDCs | 1 | (x) |  | (x) |  | 19 |  |  |
| Cells/protein | CD11c+ HLA-DR+ DCs | 2 | x |  |  |  | 19 |  |  |
| Cells/protein | CD3+ T cells | 2 | x |  |  |  | 27 | Explorative | Kucuka 2025 [1] |
| Cells/protein | CD4+ T cells | 2 | x |  |  |  | 27 |  |  |
| Cells/protein | CD8+ T cells | 2 | x |  |  |  | 27 |  |  |
| Cells/protein | Follicular T cells, age-dependent | 0 |  |  | x |  | 27 |  |  |
| Cells/protein | Th17, age-dependent | 1 |  |  | (x) |  | 27 |  |  |
| Cells/protein | Tregs, age dependent | 0 |  |  | x |  | 27 |  |  |
| Cells/protein | B cells, age-dependent | 0 |  |  | x |  | 27 |  |  |
| Cells/protein | Memory B cells | 2 | x |  |  |  | 27 |  |  |
| Cells/protein | Transitional B cells | 0 |  |  | x |  | 9 |  |  |
| Cells/protein | CD69 on trans B cells | 4 |  | X |  |  | 9 |  |  |
| Serum | APRIL serum | 3 |  | (x) |  |  | 26 |  |  |
| Serum | BAFF serum | 3 |  | (x) |  |  | 26 |  |  |
| Cells/protein | Monocytes, age-dependent | 1 |  |  | (x) |  | 27 |  |  |
| Cells/protein | pDCs | 2 | x |  |  |  | 27 |  |  |
| Cells/protein | Neutrophils | 2 | x |  |  |  | 27 |  |  |
| Cells/protein | NK cells, age-dependent | 0 |  |  | x |  | 27 |  |  |
| Cells/protein | CD3+ Va7.2+ CD161++ MAIT | 0 |  |  | x |  | 24 | Targeted | Hetemaki 2024a [2] |
| Cells/protein | Activated MAIT | 4 |  |  |  | x | 24 |  |  |
| Cells/protein | CD4- CD8- MAIT | 0 |  |  | x |  | 24 |  |  |
| Cells/protein | Memory, PD1+, CD69+ MAIT | 4 |  | X |  |  | 24 |  |  |
| Cells/protein | CD57+ MAIT | 2 | x |  |  |  | 24 |  |  |
| Cells/protein | Transitional B cells | 0 |  |  | x |  | 24 | Targeted | Hetemaki 2024b [3] |
| Cells/protein | Naive B cells | 0 |  |  | x |  | 24 |  |  |
| Cells/protein | Class switched IgD- B cells | 4 |  | X |  |  | 24 |  |  |
| Cells/protein | CD24- D38- B cells | 4 |  | X |  |  | 24 |  |  |
| Cells/protein | CD69 within B cells | 4 |  | X |  |  | 24 |  |  |
| Cells/protein | CD25 within B cells | 4 |  | X |  |  | 24 |  |  |
| Cells/protein | Circ. T follicular helper cells CXCR5 | 0 |  |  | x |  | 24 |  |  |
| Cells/protein | Circ. Tregs non-Tfh FOXP3+ CD25++ CD127- | 2 | x |  |  |  | 24 |  |  |
| Cells/protein | Circ. Tregs Tfh FOXP3+ CXCR5+ | 0 |  |  | x |  | 24 |  |  |
| Cells/protein | CTLA4 in circ. Tfh Treg | 2 | x |  |  |  | 24 |  |  |
| Serum | BAFF og IL21 | 2 | x |  |  |  | 24 |  |  |
| Serum | APRIL | 4 |  | X |  |  | 24 |  |  |
| Cells/protein | All T cells CD3+ | 4 |  | X |  |  | 19 | Explorative | Humbert 2024 [4] |
| Cells/protein | CD4+ T cells | 4 |  | X |  |  | 19 |  |  |
| Cells/protein | CD8+ T cells | 2 | x |  |  |  | 19 |  |  |
| Cells/protein | NK cells | 0 |  |  | x |  | 19 |  |  |
| Cells/protein | B cells | 0 |  |  | x |  | 19 |  |  |
| Cells/protein | Naïve B cells | 0 |  |  | x |  | 19 |  |  |
| Cells/protein | Switched memory B cells | 1 |  |  | (x) |  | 19 |  |  |
| Cells/protein | Non-switched memory B | 2 | x |  |  |  | 19 |  |  |
| Cells/protein | Suppressive capacity Tregs. expanded Tregs | 2 | x |  |  |  | 15 | Targeted | Sjøgren 2024 [5] |
| Cells/protein | CD4+CD25+FOXP3+. expanded Tregs | 0 |  |  | x |  | 17 |  |  |
| Cells/protein | Exhausted Tregs. expanded Tregs | 4 |  | X |  |  | 17 |  |  |
| Transcriptomic | TCR repertoire. expanded Tregs | 0 |  |  |  | (x) | 9 |  |  |
| Transcriptomic | B cells | 0 |  |  | x |  | 23 | Exploratory | Oftedal 2023 [6] |
| Transcriptomic | Naïve B cells | 0 |  |  | x |  | 16 |  |  |
| Transcriptomic | Mature B cells | 2 | x |  |  |  | 16 |  |  |
| Transcriptomic | Mature B cells able to switch | 0 |  |  | x |  | 16 |  |  |
| Transcriptomic | Mature B cells class switched | 0 |  |  | x |  | 16 |  |  |
| Transcriptomic | Naïve CD4+ T cells (high. not sign) | 3 | (x) | (x) |  |  | 16 |  |  |
| Transcriptomic | Central memory CD4+ T cells | 4 |  | X |  |  | 16 |  |  |
| Transcriptomic | Effector memory CD4+ T cells | 2 | x |  |  |  | 16 |  |  |
| Transcriptomic | Naïve CD8+ T cells | 2 | x |  |  |  | 16 |  |  |
| Transcriptomic | Central memory CD8+ T cells | 2 | x |  |  |  | 16 |  |  |
| Transcriptomic | Effector memory CD8+ T cells (low. not sign) | 1 | (x) |  |  |  | 16 |  |  |
| Transcriptomic | NK (low. not sign.) | 1 | (x) |  |  |  | 16 |  |  |
| Transcriptomic | NKT | 2 | x |  |  |  | 16 |  |  |
| Transcriptomic | Granulocytes | 4 |  | X |  |  | 16 |  |  |
| Transcriptomic | pDCs (low. not sign.) | 1 | (x) |  |  |  | 16 |  |  |
| Transcriptomic | Myeloid DC | 2 | x |  |  |  | 16 |  |  |
| Transcriptomic | Monocytes | 4 |  | X |  |  | 16 |  |  |
| Cells/protein | Transitional B | 2 | x |  |  |  | 15 | Targeted | Sng 2019 [7] |
| Cells/protein | Follicular T cells | 2 | x |  |  |  | 13 |  |  |
| Cells/protein | CD69+ Mature naive B celler | 4 |  | X |  |  | 12 |  |  |
| Cells/protein | Tregs repertoire | 0 |  |  |  | x | 12 |  |  |
| Cells/protein | Tregs HLA-DR+ | 0 |  |  | x |  | 12 |  |  |
| Cells/protein | Tregs FOXP3 and CD127- | 0 |  |  | x |  | 12 |  |  |
| Cells/protein | Circulating. autoreactive. mature B | 4 |  | X |  |  | 12 |  |  |
| Cells/protein | Mature naive B increased prolif | 4 |  |  |  | x | 12 |  |  |
| Serum | IFNg. IL2. GM-CSF. IL4. IL5. IL10 | 4 |  | X |  |  | 12 |  |  |
| Cells/protein | Tregs CD4+CD25hiCD127-/FOXP3+/Helios | 4 |  |  | x |  | 12 |  |  |
| Cells/protein | polyclonal new emigrant/transitional B | 2 | x |  |  |  | 12 |  |  |
| Cells/protein | Ig H-chain repertoire | 2 | x |  |  |  | 12 |  |  |
| Cells/protein | CCR6+ CXCR3- Th17+Th22 | 4 |  | X |  |  | 11 |  |  |
| Cells/protein | CCR6- CXCR3+ Th1 | 0 |  |  | x |  | 11 |  |  |
| Cells/protein | PMA-activation CD4+ IFNg.TNFa.IL2.Tbet | 0 |  |  | x |  | 12 |  |  |
| Cells/protein | PMA-activation CD8+ IFNg.TNFa.IL2.Tbet | 0 |  |  | x |  | 12 |  |  |
| Cells/protein | PMA-activation γδT IFNg.TNFa.IL2.Tbet | 0 |  |  | x |  | 12 |  |  |
| Cells/protein | Suppression Tregs | 0 |  |  | x |  | 8 |  |  |
| Serum | IFNg. IL2. GM-CSF. IL4. IL5. IL10 | 4 |  | X |  |  | 8 |  |  |
| Cells/protein | TCR repertoire TCRBV conventional T. TCR diversitet | 2 | x |  |  |  | 5 |  |  |
| Cells/protein | Expression of HEp-2-antibodies (B cells) | 4 |  | X |  |  | 4 |  |  |
| Cells/protein | B cells | 0 |  |  | x |  | 25 | Targeted | Perri 2017 [8] |
| Cells/protein | Mature B cells CD27- | 2 | x |  |  |  | 25 |  |  |
| Cells/protein | Memory B cells CD27+ IgM+ | 4 |  | X |  |  | 25 |  |  |
| Cells/protein | Transitional B cells CD27- CD24hi CD38+ | 2 | x |  |  |  | 25 |  |  |
| Cells/protein | Switched memory B CD19+ CD27+ IgM- | 0 |  |  | x |  | 25 |  |  |
| Cells/protein | Stim. B 4 days prolif. Response | 0 |  |  |  | x | 16 |  |  |
| Cells/protein | Stim. B 4 days Mature B cells | 0 |  |  |  | X | 16 |  |  |
| Cells/protein | Stim. B 4 days switched memory | 2 | x |  |  |  | 16 |  |  |
| Cells/protein | Stim. B 7 days. No difference B cells | 2 | x |  |  |  | 21 |  |  |
| Cells/protein | Stim. B 7 days. Production of antibodies (IgA, IgG, IgM) | 4 |  |  |  | x | 12 |  |  |
| Cells/protein | CD4+ T cells | 4 |  | X |  |  | 35 | Exploratory | Ferre 2016 [9] |
| Cells/protein | CD4/CD8 T cells | 4 |  | X |  |  | 35 |  |  |
| Cells/protein | CD3+ T cells | 4 |  | X |  |  | 35 |  |  |
| Cells/protein | Naïve CD4+ | 0 |  |  | x |  | 35 |  |  |
| Cells/protein | Central memory CD8+ | 0 |  |  | x |  | 35 |  |  |
| Cells/protein | Other T cell compartments | 2 | x |  |  |  | 35 |  |  |
| Cells/protein | B cells | 2 | x |  |  |  | 30 |  |  |
| Cells/protein | Plasmablasts | 0 |  |  | x |  | 30 |  |  |
| Cells/protein | CD21low CD38low B cells | 4 |  | X |  |  | 30 |  |  |
| Cells/protein | NK cells | 0 |  |  | x |  | 35 |  |  |
| Cells/protein | NKT cells | 1 | (x) | (x) |  |  | 35 |  |  |
| Cells/protein | CD4+. CD8+. CD4:CD8+ ratio | 2 | x |  |  |  | 10 | Targeted | Heikkila 2016 [10] |
| Cells/protein | CD4+ effector/memory T | 4 |  | X |  |  | 10 |  |  |
| Cells/protein | Naive CD4+ CCR7+ | 0 |  |  | x |  | 10 |  |  |
| Cells/protein | CD8+ CD45RA+ CCR7- effector | 0 |  |  | x |  | 10 |  |  |
| Cells/protein | Th subclasses | 2 | x |  |  |  | 10 |  |  |
| Cells/protein | Expression of Th1. Th2. Th17 cytokines |  |  | X | x |  | 7 |  |  |
| Cells/protein | Memory cells: Expression of IFNg after anti-CD3 | 0 |  |  | x |  | 7 |  |  |
| Cells/protein | Expression of IL4 and IL17 after anti-CD3 stimulus | 2 | x |  |  |  | 7 |  |  |
| Transcriptomic | T: IFNg pathway abnormalities |  |  | X | x |  | 3 |  |  |
| Cells/protein | CD4+ RTE | 0 |  |  | x |  | 3 |  |  |
| Cells/protein | IL7 levels T cells | 4 |  | X |  |  | 12 | Targeted | Laakso 2011 [11] |
| Cells/protein | IL7R on CD8+ T cells | 0 |  |  | x |  | 12 |  |  |
| Cells/protein | Tregs RTE turnover | 4 |  | X |  |  | 12 | Targeted | Laakso 2010 [12] |
| Cells/protein | Activated Treg pool less FOXP3 | 0 |  |  | x |  | 12 |  |  |
| Cells/protein | Tregs CD4+ CD25hi FOXP3 | 0 |  |  | x |  | 19 | Explorative | Wolff 2010 [13] |
| Cells/protein | Tregs CD4+ CD25hi CD127- | 0 |  |  | x |  | 19 |  |  |
| Cells/protein | NKT Va24. Vb11. CD161. iNKT | 2 | x |  |  |  | 19 |  |  |
| Cells/protein | CD8+ CD11b+ CD28+ | 2 | x |  |  |  | 19 |  |  |
| Cells/protein | CD4+ CCR6+ CXCR3+ (IFNg+IL17A) | 0 |  |  | x |  | 19 |  |  |
| Cells/protein | CD3+CD4+ T cells | 2 | x |  |  |  | 19 |  |  |
| Cells/protein | CD3+CD8+ T cells | 2 | x |  |  |  | 19 |  |  |
| Cells/protein | CD3 T cells | 2 | x |  |  |  | 19 |  |  |
| Cells/protein | CD4+ CCR4+CCR6+ (Th17) | 2 | x |  |  |  | 19 |  |  |
| Cells/protein | CD14 monocytes | 2 | x |  |  |  | 19 |  |  |
| Cells/protein | CD14+ CD16+ | 4 |  | X |  |  | 19 |  |  |
| Cells/protein | DC and DC subtypes | 2 | x |  |  |  | 19 |  |  |
| Cells/protein | B cells CD19+ | 2 | x |  |  |  | 19 |  |  |
| Cells/protein | NK cells CD3- D56+ | 2 | x |  |  |  | 19 |  |  |
| Cells/protein | CD5+ B cells | 2 | x |  |  |  | 19 |  |  |
| Cells/protein | pDCs | 2 | x |  |  |  | 13 | Targeted | Hong 2009 [14] |
| Cells/protein | mDCs | 2 | x |  |  |  | 13 |  |  |
| Cells/protein | Monocytes | 0 |  |  | x |  | 13 |  |  |
| Cells/protein | TLR2+ and TLR6+ monocytes | 2 | x |  |  |  | 13 |  |  |
| Cells/protein | TLR1. TLR4. TLR6 failed to downreg. After stimulus | 4 |  | X |  |  | 13 |  |  |
| Cells/protein | TLR2. TLR6 failed to downreg. After stimulus | 4 |  | X |  |  | 13 |  |  |
| Cells/protein | TLR-3. TLR5. TLR10 reaction to stimuli | 2 | x |  |  |  | 13 |  |  |
| Cells/protein | Dectin-1. Syk. CARD9 reaction to stimuli | 4 |  | X |  |  | 13 |  |  |
| Cells/protein | γδ T cells | 2 | x |  |  |  | 24 | Targeted | Tuovinen 2009 [15] |
| Serum | IgG | 4 |  | X |  |  | 11 | Targeted | Perniola 2008 [16] |
| Serum | IgA. IgM | 2 | x |  |  |  | 11 |  |  |
| Cells/protein | Monocytes | 4 |  | X |  |  | 11 |  |  |
| Cells/protein | Suppressive capacity CD4+CD25+ | 0 |  |  | x | x | 24 | Targeted | Kekalainen 2007 [17] |
| Cells/protein | CD4+ CD25+ CD62L+ | 2 | x |  |  |  | 20 |  |  |
| Cells/protein | CD4+ CD25+ HLA-DR+ | 2 | x |  |  |  | 14 |  |  |
| Transcriptomic | TCRbeta repertoire | 4 |  | X |  |  | 14? |  |  |
| Cells/protein | CD4+CD25hi | 2 | x |  |  |  | 11 |  |  |
| Cells/protein and RNA | CD4+ CD25hi FOXP3+ | 0 |  |  | x |  | 10 |  |  |
| Cells/protein and RNA | CD4+ CD25lo FOXP3+ | 0 |  |  | x |  | 10 |  |  |
| Cells/protein | CD4+ CD25+ CD45RO+ | 2 | x |  |  |  | 6 |  |  |
| Cells/protein | Candida-proliferation resp. to C.albicans | 2 | x |  |  |  | 5 |  |  |
| Cells/protein | TCR excision circle content of blood | 2 | x |  |  |  | NA |  |  |
| Transcriptomic | CDR3 lengths | 4 |  | X |  |  | 1? |  |  |
| Cells/protein | CD3+ T cells | 4 |  | X |  |  | 11 | Explorative | Perniola 2005 [18] |
| Cells/protein | CD3+ CD4+ | 4 |  | X |  |  | 11 |  |  |
| Cells/protein | CD3+ CD8+ | 2 | x |  |  |  | 11 |  |  |
| Cells/protein | Tregs CD4+ CD25+ | 0 |  |  | x |  | 11 |  |  |
| Cells/protein | CD19+ B cells | 2 | x |  |  |  | 11 |  |  |
| Cells/protein | CD3- CD16/CD56+ NK | 2 | x |  |  |  | 11 |  |  |
| Cells/protein | activated T cells (CD25+. CD69. HLA-DR+. CD45RO+) | 4 |  | X |  |  | 11 |  |  |
| Cells/protein | CD8+CD11b+ suppressor cells | 0 |  |  | x |  | 11 |  |  |
| Cells/protein | CD3- CD8+ | 0 |  |  | x |  | 11 |  |  |

1. Studies of immune cell compositions in APS-1 (smaller studies. N<10)

| **Level** | **Immune molecule/subset** | **score** | **Normal** | **Up** | **Down** | **Functional flaws** | **# APS-1** | **Study** | **Ref** |
| --- | --- | --- | --- | --- | --- | --- | --- | --- | --- |
| Transcriptomic | Tregs metabolic disturbances |  |  | x | x | x | 9 | Targeted | Berger 2021[19] |
| Protein | CD4+ |  | x |  |  |  | 5 |  |  |
| Transcriptomic | Tregs (a little low) |  | (x) |  | (x) |  | 5 |  |  |
| Transcriptomic | Markers within Tregs CD31. CD39. CD45RA. Ki67 |  | x |  |  |  | 5 |  |  |
| Transcriptomic | Markers within Tregs: Helios a little low |  |  |  | x |  | 5 |  |  |
| Cells/protein | γδ T cells |  | x |  |  |  | 8 | Targeted | Kaleviste 2020[20] |
| Cells/protein | NKT cells (low. not sign) |  | (x) |  | (x) |  | 8 |  |  |
| Cells/protein | MAIT-cells |  |  |  | x |  | 8 |  |  |
| Cells/protein | TCR beta CD3 length distribution |  | x |  |  |  | 5 | Targeted | Niemi 2015[21] |
| Cells/protein | CD4+ Th response |  | x |  |  |  | 4 | Targeted | Laakso 2014[22] |
| Transcriptomic | Th1 and Th2 responses |  | x |  |  |  | 3 |  |  |
| Cells/protein | NKT |  |  |  | x |  | 3 | Targeted | Lindh 2010[23] |
| Cells/protein | NK |  | x |  |  |  | 2 |  |  |
| Cells/protein | pDC (low. not sign.) |  | (x) | (x) |  |  | 5 | Targeted | Kisand 2008[24] |
| Cells/protein | VCAM+ PBMCs |  |  | x |  |  | 4 | Targeted | Ramsey 2006[25] |
| Cells/protein | VCAM+ monocytes |  |  | x |  |  | 4 |  |  |
| Cells/protein | Monocytes |  |  | x |  |  | 4 |  |  |
| Cells/protein | IgG. IgA. C3. C4. IgG subclasses |  | x |  |  |  | 4 | Explorative | Sediva 2002[26] |
| Cells/protein | IgM in some |  |  | x |  |  | 4 |  |  |
| Cells/protein | CD3+ CD4+ in some |  |  |  | x |  | 4 |  |  |
| Cells/protein | B cells. T cells. their subtypes |  | x |  |  |  | 4 |  |  |
| Cells/protein | Th1 and Th2 cytokines. IFN-γ |  |  |  | x | x | 4 |  |  |
| Serum | IgG. IgM |  | x |  |  |  | 2 | Explorative | Ishii 2000[27] |
| Serum | IgA |  |  |  | x |  | 2 |  |  |
| Cells/protein | CD4+ and CD8+ T cells |  | x |  |  |  | 2 |  |  |
| Cells/protein | Neutrophil count |  | x |  |  |  | 2 |  |  |
| Cells/protein | Suppressor T cells |  |  |  | x |  | 5 | Targeted | O'Sullivan 1997[28] |
| Serum | IgM |  |  | x |  |  | 3 | Targeted | Arulanantham 1979[29] |
| Serum | IgE |  |  | x |  |  | 3 |  |  |
| Serum | IgG |  |  | x |  |  | 3 |  |  |
| Serum | IgA in some |  |  |  | x |  | 3 |  |  |
| Cells/protein | Suppressor T cells |  |  |  | x | x | 3 |  |  |

Score: 0 is low. 1 is a little low. 2 is neutral. 3 is a little high. 4 is high level in APS-1patients compared to healthy controls.

Supplementary Figure 1 (Fig. S1)


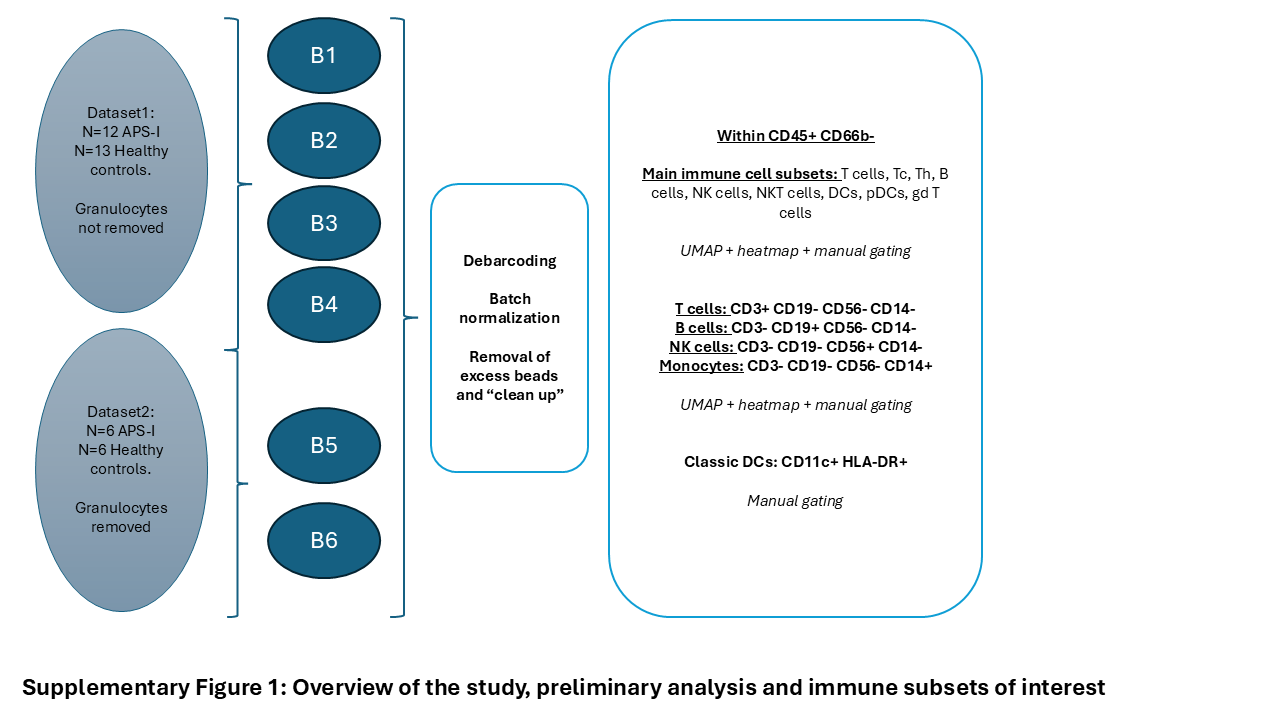


Legend: Overview of study. APS-I: Autoimmune polyendocrine syndrome type I (APS-I); HC: Healthy controls; B: Cytof batch number; NK: Natural killer; DC: Dendritic cells; pDC: plasmocytoid dendritic cells; γδ: gamma delta.

Supplementary Figure 2 (Fig. S2)


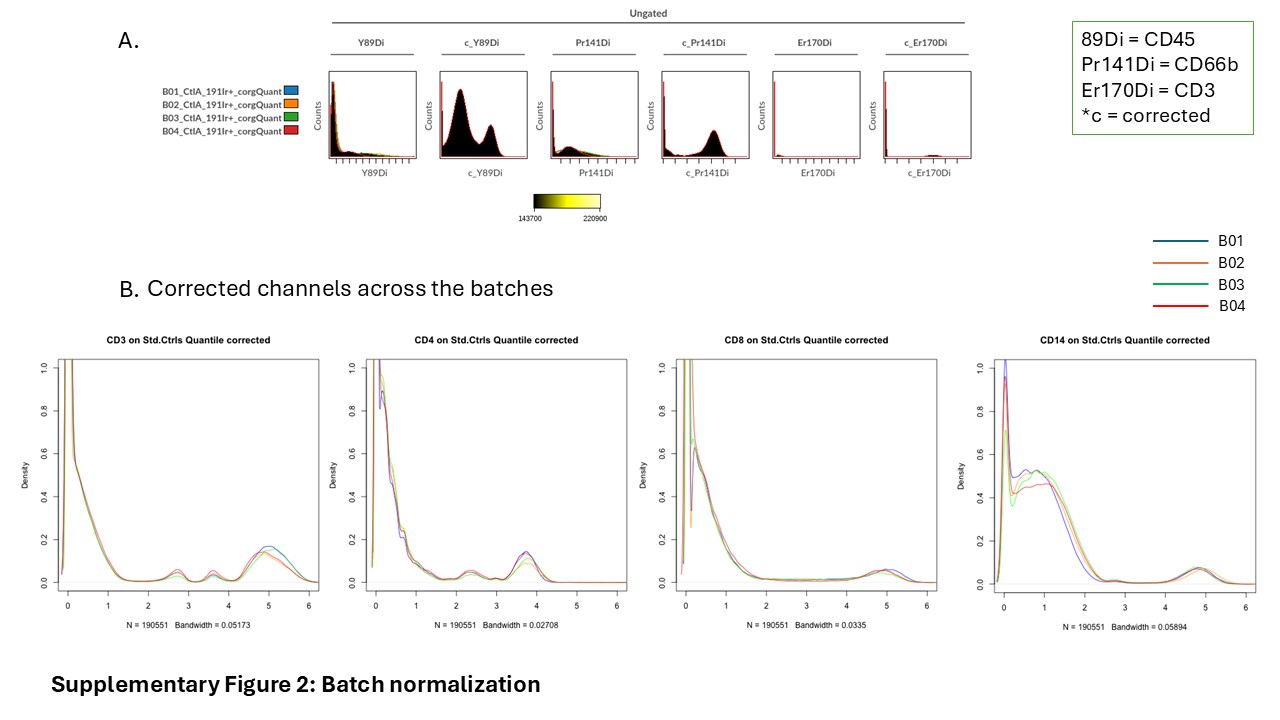


Legend: Batch correction algorithm testing (Dataset 1). All plots were generated from normalized arcsinh-transformed CD45+CD66b- cells. The transformed datasets were quantile corrected. (A) Biaxial histogram from the four testing batches with exemplified lineage markers (CD45. CD66b. and CD3). The files displayed are the first uncorrected. then corrected (‘C’ as the prefix) on the internal control sample from each batch. (B) Cydar batch correction density plots of four representative markers (CD3. CD4. CD8. and CD14) showing each of the four experimental batches.

Supplementary Figure 3 (Fig. S3)


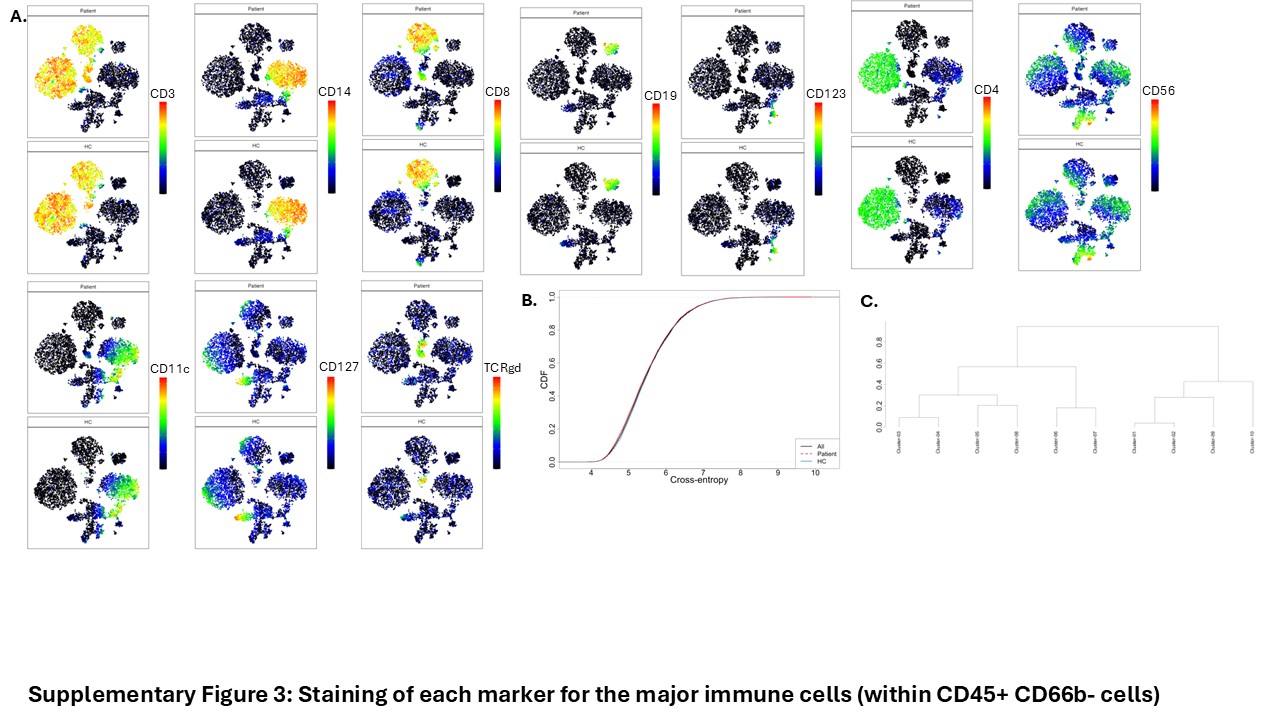


Legend: Details of the unsupervised gating of all major immune cells from Dataset 1. A. The intensity of individual (lineage) markers are displayed in individual t-SNE plots (FlowSOM) for the major immune cells (CD45+CD66-) subsets in autoimmune polyendocrine syndrome type I (APS-I) patients and healthy controls (HC) (Dataset 1). The heat scale is shown.

Supplementary Fig. 4 (Fig. S4)


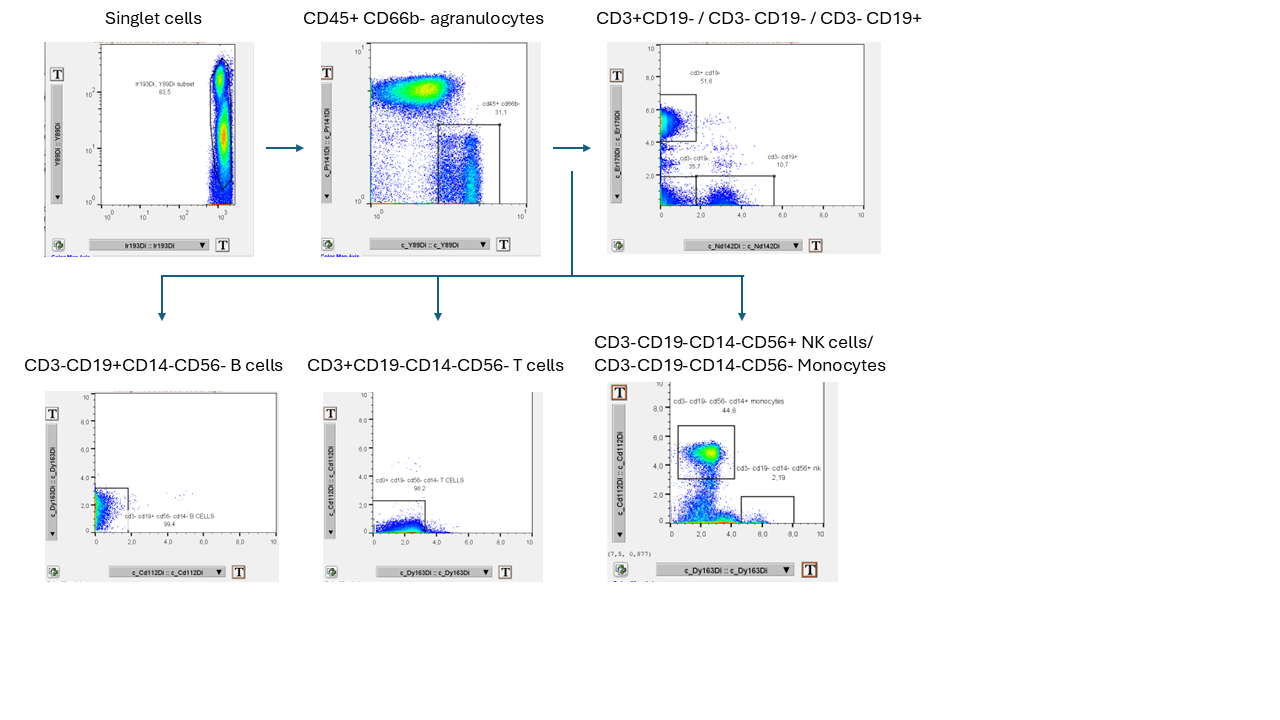


**Supplementary Figure 4: Gating on B cells, T cells, NK cells and Monocytes.**

Legend: The figure shows fcs.file-data on the single cell level, and the gating strategy for the main immune cell subpoulations, in FlowJo (CD45+ CD66b-, CD3- CD19+CD56-CD14- B cells, CD3+CD19-CD56-CD14- T cells, CD3-CD19-CD56+CD14- NK cells and CD3-CD19-CD56-CD14+ Monocytes).

**
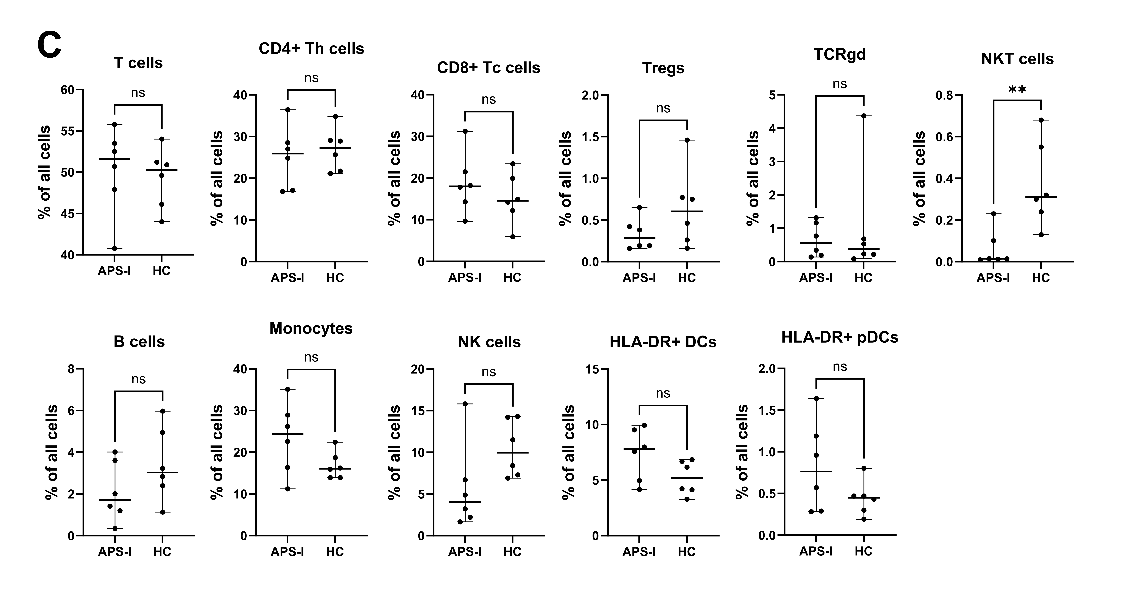
**Supplementary Fig. 5 (Fig. S5)

**B**


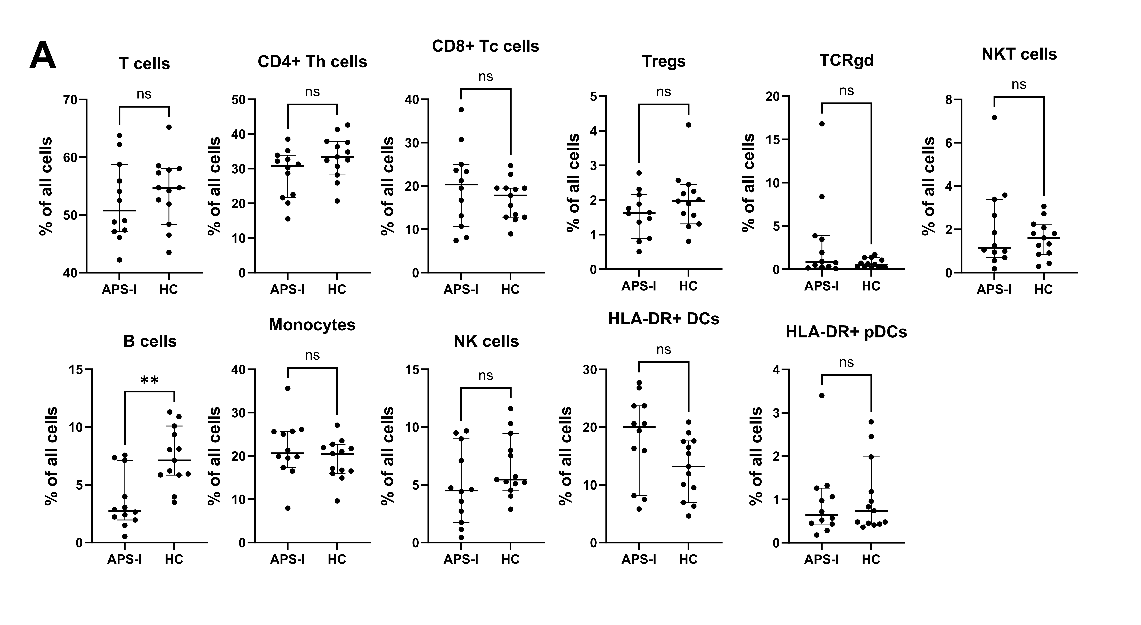


**Supplementary Figure 5. Analysis of Dataset 1 and 2 separately on manually gated CD45**+ **CD66b- cells.**

Legend: The graphs illustrate the percentage of events in the manually gated major (lineage) immune cell subsets with respect to CD45+CD66b- (all cells; 1611173 from healthy controls and 1577967 from APS-I patients) among APS-I patients and healthy controls (HC). Mann-Whitney tests were performed for statistical evaluations for each dataset in Prism Graphpad v. 10 (P<0.05). The X-axis shows the percentage of all cells, while the Y-axis represents the population. P-values are shown in each graph/for each population: ns (non-significant): p>0.01, 0.01>p<0.05 *, p<0.001 **. A. The plots show the distribution of events in the designated immune subsets in Dataset 1. B. The plots show the distribution of events in the designated immune subsets in Dataset 2. Median and 95% confidence interval are shown for each graph.

Supplementary Fig. 6 (Fig. S6)


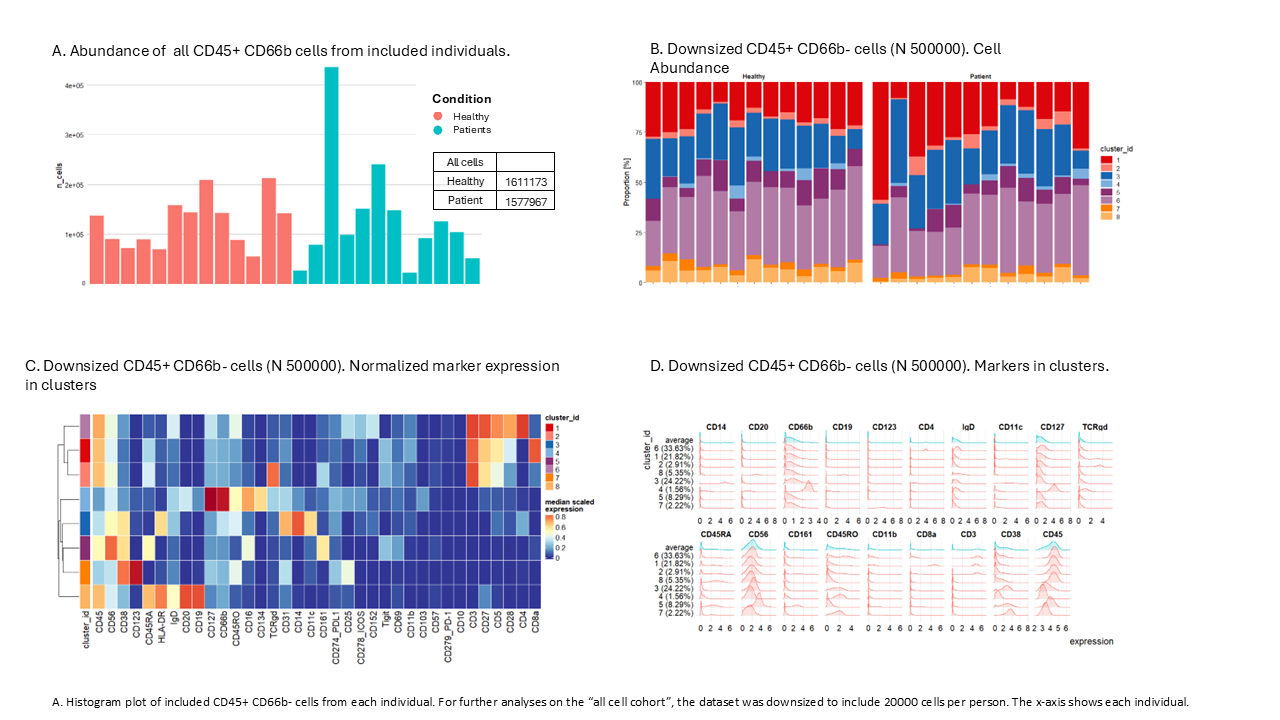


**Supplementary Figure 6:** **Supporting data from analysis of CD45+ CD66b- cells from APS-I patients and healthy controls (Dataset 1)**

Legend: Twenty thousand CD45+ CD66b- cells from each of the 25 included individuals were included (original N=1611173 cells from healthy controls and N=1577967 cells from APS-I patients). A. Cell number from each individual. B. The proportion of cells in the 8 clusters of each individual separated by healthy controls and patients. The cluster identity is shown on the right side of the graph. C. Heatmap of normalized marker intensities in each cluster. The cluster identity and expression scale is shown on the right side of the graph. D. Marker intensity in each of the eight clusters shown by a wave plot.

Supplementary Figure 7 (Fig. S7)

**
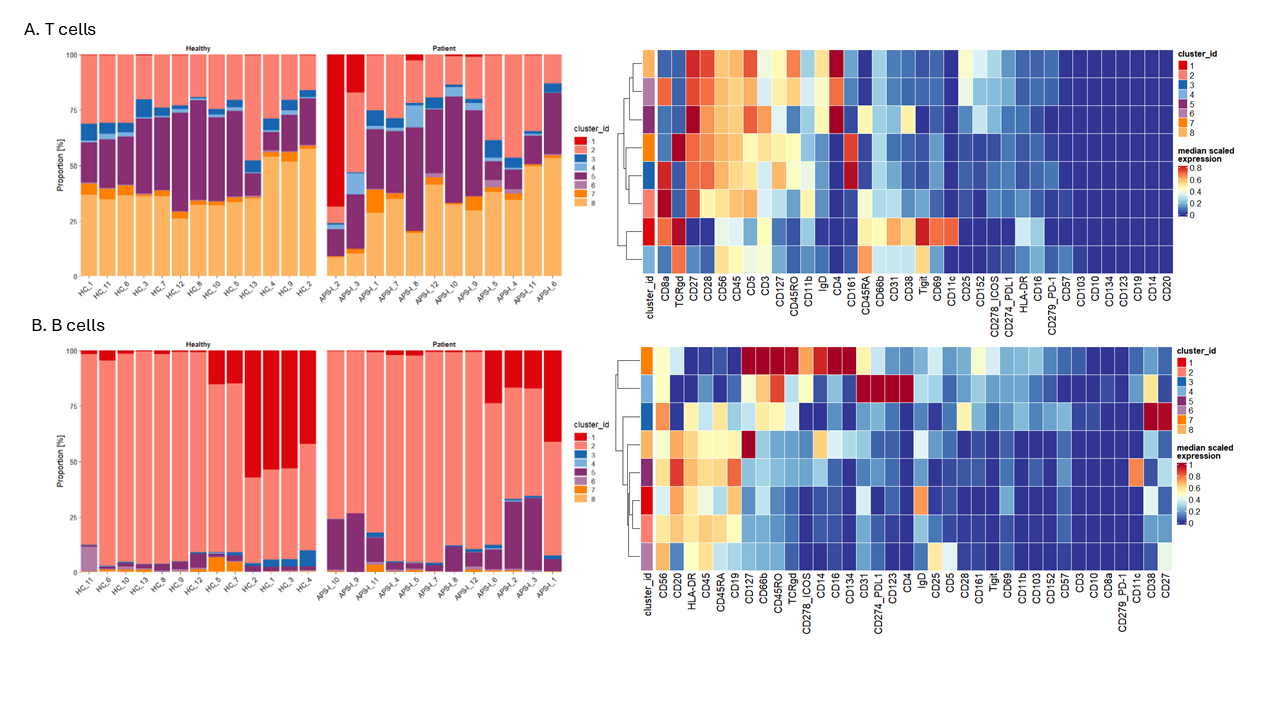
**

**
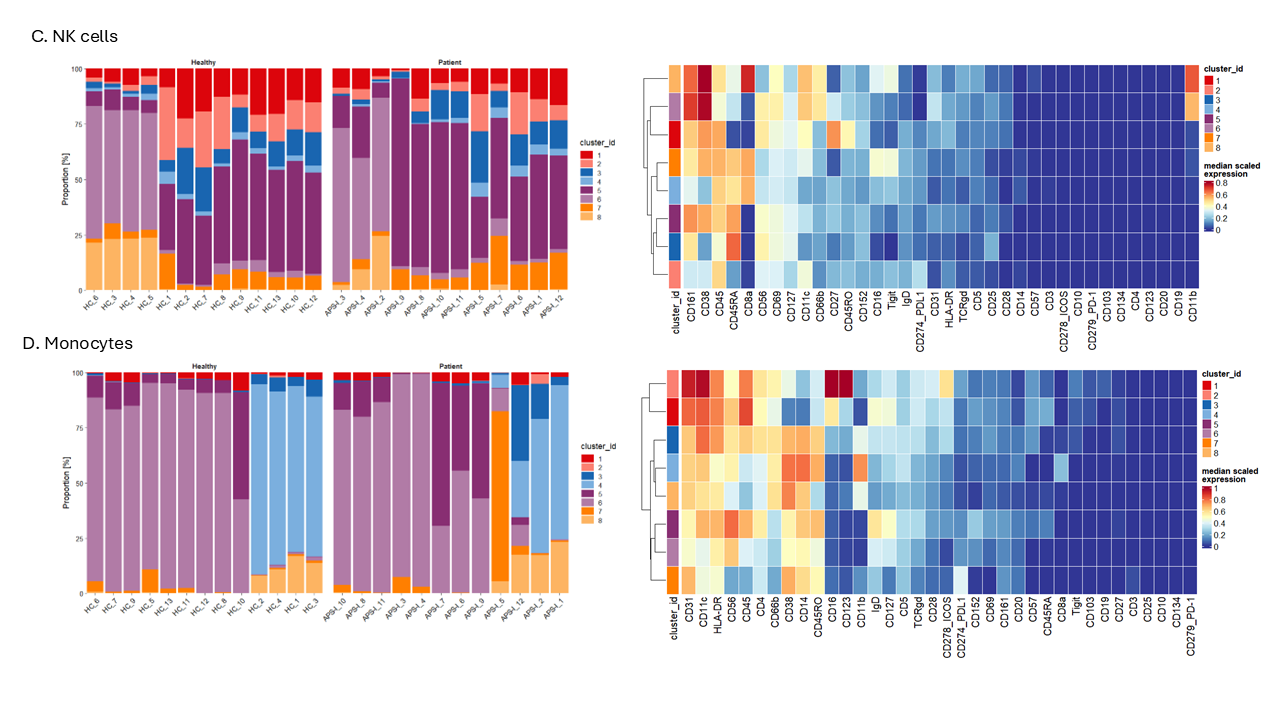
**

**Supplementary Figure 7 Abundance plot and marker heatmap for immune subpopulations from APS-I patients and healthy controls (Dataset 1).**

Legend: All cells within the CD45+ CD66b- immune subpopulations of interest from each of the 25 included individuals were included. For each panel there is an abundance plot, viewing the proportion of cells in the eight clusters of each individual separated by healthy controls and patients, and a heatmap of normalized marker intensities in each cluster. A. CD3+ CD19- CD56- CD14- T cells, B. CD3- CD19+ CD56- CD14- B cells, C. CD3- D19- CD56+ CD14- NK cells, D. CD3- CD19- CD56- CD14+ Monocytes. The cluster identity and expression scale are shown on the right side of the plots.

**References Supplementary material**

[1] I. Kucuka, D. Iraji, S. Braun, L. Breivik, A. S. B. Wolff, E. S. Husebye *et al.* Longitudinal Immune Profiling in Autoimmune Polyendocrine Syndrome Type 1. Scand J Immunol, 2025;101**:**e70021.

[2] I. Hetemaki, J. Sarkkinen, H. H. Wong, N. Heikkila, S. Laakso, S. Miettinen *et al.* Reduction in mucosal-associated invariant T cells (MAIT) in APECED patients is associated with elevated serum IFN-gamma concentration. Eur J Immunol, 2024;54**:**e2451189.

[3] I. Hetemaki, J. Sarkkinen, N. Heikkila, K. Drechsel, M. I. Mayranpaa, A. Farkkila *et al.* Dysregulated germinal center reaction with expanded T follicular helper cells in autoimmune polyendocrinopathy-candidiasis-ectodermal dystrophy lymph nodes. J Allergy Clin Immunol, 2024;153**:**1445-55.

[4] L. Humbert, E. Proust-Lemoine, S. Dubucquoi, E. H. Kemp, P. Saugier-Veber, N. Fabien *et al.* Lessons from prospective longitudinal follow-up of a French APECED cohort. J Clin Endocrinol Metab, 2024.

[5] S. I. Thea Sjøgren, Igor Filippov#, Adrianna Jebrzycka, André Sulen, Lars E. Breivik, Alexander Hellesen, Anders P. Jørgensen, Kari Lima, Liina Tserel, Kai Kisand, Pärt Peterson, Annamari Ranki, Eystein S. Husebye, Bergithe E. Oftedal, Anette S. B. Wolff Single cell characterization of blood and expanded regulatory T cells in autoimmune polyendocrine syndrome type 1. iScience, 2024.

[6] N. D. Bergithe E. Oftedal, David Dolan, Anthony Meager, Eystein S. Husebye, Anette S. B. Wolff. Mild decrease in systemic interferon type I and B cell regulation responses in autoimmune polyendocrine syndrome type 1. FEBS Letters, 2023;Accepted for publication 24th March 2023.

[7] J. Sng, B. Ayoglu, J. W. Chen, J. N. Schickel, E. M. N. Ferre, S. Glauzy *et al.* AIRE expression controls the peripheral selection of autoreactive B cells. Sci Immunol, 2019;4.

[8] V. Perri, E. Gianchecchi, R. Scarpa, M. Valenzise, M. M. Rosado, E. Giorda *et al.* Altered B cell homeostasis and Toll-like receptor 9-driven response in patients affected by autoimmune polyglandular syndrome Type 1: Altered B cell phenotype and dysregulation of the B cell function in APECED patients. Immunobiology, 2017;222**:**372-83.

[9] E. M. Ferre, S. R. Rose, S. D. Rosenzweig, P. D. Burbelo, K. R. Romito, J. E. Niemela *et al.* Redefined clinical features and diagnostic criteria in autoimmune polyendocrinopathy-candidiasis-ectodermal dystrophy. JCI Insight, 2016;1.

[10] N. Heikkila, S. M. Laakso, H. Mannerstrom, E. Kekalainen, P. Saavalainen, H. Jarva *et al.* Expanded CD4(+) Effector/Memory T Cell Subset in APECED Produces Predominantly Interferon Gamma. J Clin Immunol, 2016;36**:**555-63.

[11] S. M. Laakso, E. Kekalainen, L. H. Rossi, T. T. Laurinolli, H. Mannerstrom, N. Heikkila *et al.* IL-7 dysregulation and loss of CD8+ T cell homeostasis in the monogenic human disease autoimmune polyendocrinopathy-candidiasis-ectodermal dystrophy. J Immunol, 2011;187**:**2023-30.

[12] S. M. Laakso, T. T. Laurinolli, L. H. Rossi, A. Lehtoviita, H. Sairanen, J. Perheentupa *et al.* Regulatory T cell defect in APECED patients is associated with loss of naive FOXP3(+) precursors and impaired activated population. J Autoimmun, 2010;35**:**351-7.

[13] A. S. Wolff, B. E. Oftedal, K. Kisand, E. Ersvaer, K. Lima, E. S. Husebye. Flow cytometry study of blood cell subtypes reflects autoimmune and inflammatory processes in autoimmune polyendocrine syndrome type I. Scand J Immunol, 2010;71**:**459-67.

[14] M. Hong, K. R. Ryan, P. D. Arkwright, A. R. Gennery, C. Costigan, M. Dominguez *et al.* Pattern recognition receptor expression is not impaired in patients with chronic mucocutanous candidiasis with or without autoimmune polyendocrinopathy candidiasis ectodermal dystrophy. Clin Exp Immunol, 2009;156**:**40-51.

[15] H. Tuovinen, N. Pontynen, M. Gylling, E. Kekalainen, J. Perheentupa, A. Miettinen *et al.* gammadelta T cells develop independently of Aire. Cell Immunol, 2009;257**:**5-12.

[16] R. Perniola, M. Congedo, A. Rizzo, A. Sticchi Damiani, M. L. Faneschi, M. Pizzolante *et al.* Innate and adaptive immunity in patients with autoimmune polyendocrinopathy-candidiasis-ectodermal dystrophy. Mycoses, 2008;51**:**228-35.

[17] E. Kekalainen, A. Miettinen, T. P. Arstila. Does the deficiency of Aire in mice really resemble human APECED? Nat Rev Immunol, 2007;7**:**1.

[18] R. Perniola, G. Lobreglio, M. C. Rosatelli, E. Pitotti, E. Accogli, C. De Rinaldis. Immunophenotypic characterisation of peripheral blood lymphocytes in autoimmune polyglandular syndrome type 1: clinical study and review of the literature. J Pediatr Endocrinol Metab, 2005;18**:**155-64.

[19] A. H. Berger, E. Bratland, T. Sjogren, M. Heimli, T. Tyssedal, O. Bruserud *et al.* Transcriptional Changes in Regulatory T Cells From Patients With Autoimmune Polyendocrine Syndrome Type 1 Suggest Functional Impairment of Lipid Metabolism and Gut Homing. Front Immunol, 2021;12**:**722860.

[20] E. Kaleviste, M. Ruhlemann, J. Karner, L. Haljasmagi, L. Tserel, E. Org *et al.* IL-22 Paucity in APECED Is Associated With Mucosal and Microbial Alterations in Oral Cavity. Front Immunol, 2020;11**:**838.

[21] H. J. Niemi, S. Laakso, J. T. Salminen, T. P. Arstila, A. Tuulasvaara. A normal T cell receptor beta CDR3 length distribution in patients with APECED. Cell Immunol, 2015;295**:**99-104.

[22] S. M. Laakso, E. Kekalainen, N. Heikkila, H. Mannerstrom, K. Kisand, P. Peterson *et al.* In vivo analysis of helper T cell responses in patients with autoimmune polyendocrinopathy - candidiasis - ectodermal dystrophy provides evidence in support of an IL-22 defect. Autoimmunity, 2014;47**:**556-62.

[23] E. Lindh, E. Rosmaraki, L. Berg, H. Brauner, M. C. Karlsson, L. Peltonen *et al.* AIRE deficiency leads to impaired iNKT cell development. J Autoimmun, 2010;34**:**66-72.

[24] K. Kisand, M. Link, A. S. Wolff, A. Meager, L. Tserel, T. Org *et al.* Interferon autoantibodies associated with AIRE deficiency decrease the expression of IFN-stimulated genes. Blood, 2008;112**:**2657-66.

[25] C. Ramsey, S. Hassler, P. Marits, O. Kampe, C. D. Surh, L. Peltonen *et al.* Increased antigen presenting cell-mediated T cell activation in mice and patients without the autoimmune regulator. Eur J Immunol, 2006;36**:**305-17.

[26] A. Sediva, D. Cihakova, J. Lebl. Immunological findings in patients with autoimmune polyendocrinopathy-candidiasis-ectodermal dystrophy (APECED) and their family members: are heterozygotes subclinically affected? J Pediatr Endocrinol Metab, 2002;15**:**1491-6.

[27] T. Ishii, Y. Suzuki, N. Ando, N. Matsuo, T. Ogata. Novel mutations of the autoimmune regulator gene in two siblings with autoimmune polyendocrinopathy-candidiasis-ectodermal dystrophy. J Clin Endocrinol Metab, 2000;85**:**2922-6.

[28] D. J. O'Sullivan, C. Cronin, D. Buckley, T. Mitchell, D. Jenkins, J. Greally *et al.* Unusual manifestations of type 1 autoimmune polyendocrinopathy. Ir Med J, 1997;90**:**101-3.

[29] K. Arulanantham, J. M. Dwyer, M. Genel. Evidence for defective immunoregulation in the syndrome of familial candidiasis endocrinopathy. N Engl J Med, 1979;300**:**164-8.

**Statistical packages and analyses: Additional references**

R-project https://www.R-project.org

r studio http://www.rstudio.com/.

Catalyst https://github.com/HelenaLC/CATALYST

Premessa <https://github.com/ParkerICI/premessa>

Cydar Lun ATL, Richard AC, Marioni JC (2017). “Testing for differential abundance in mass cytometry data.” *Nat. Methods*, **14**, 707-709. <https://www.bioconductor.org/packages/release/bioc/html/cydar.html>

ncdfFlow Gopalakrishnan MJF (2025). *ncdfFlow: ncdfFlow: A package that provides HDF5 based storage for flow cytometry data* and <https://www.bioconductor.org/packages/release/bioc/html/ncdfFlow.html>

flowSOM Van Gassen S, Callebaut B, Van Helden M, Lambrecht B, Demeester P, Dhaene T, Saeys Y (2015). “FlowSOM: Using self-organizing maps for visualization and interpretation of cytometry data.” *Cytometry Part A*, **87**(7), 636-645.  <https://onlinelibrary.wiley.com/doi/full/10.1002/cyto.a.22625>.

flowcore Ellis B, Haaland P, Hahne F, Le Meur N, Gopalakrishnan N, Spidlen J, Jiang M, Finak G (2025). *flowCore: flowCore: Basic structures for flow cytometry data* and <https://www.bioconductor.org/packages/release/bioc/html/flowCore.html>

flowcytoscript <https://github.com/DrCytometer/Simplified-flowcytoscript>.

CRAN devtools https://cran.r-project.org/web/packages/devtools/index.html

Scater McCarthy, D. J., Campbell, K. R., Lun, A. T. L., & Wills, Q. F. (2017). Scater: Pre-processing, quality control, normalization, and visualization of single-cell RNA-seq data in R. *Bioinformatics*, 33(8), 1179–1186. https://doi.org/10.1093/bioinformatics/btw777

diffCyt Weber LM, Nowicka M, Soneson C, Robinson MD (2019). “diffcyt: Differential discovery in high-dimensional cytometry via high-resolution clustering.” *Communications Biology*, **2**(183).

EdgeR Robinson MD, McCarthy DJ and Smyth GK (2010). **edgeR**: a Bioconductor **package** for differential expression analysis of digital gene expression data. Bioinformatics 26, 139-140 McCarthy DJ, Chen Y and Smyth GK (2012).

Limma Ritchie, M.E., Phipson, B., Wu, D., Hu, Y., Law, C.W., Shi, W., and Smyth, G.K. (2015). limma powers differential expression analyses for RNA-sequencing and microarray studies. Nucleic Acids Research 43(7), e47.

ggplot Wickham H (2016). *ggplot2: Elegant Graphics for Data Analysis*. Springer-Verlag New York. ISBN 978-3-319-24277-4, [https://ggplot2.tidyverse.org](https://ggplot2.tidyverse.org/).

Cytofcore <https://github.com/nolanlab/cytofCore>

StataNow 18 SE StataCorp. 2024. StataNow 18 SE. College Station, TX: StataCorp LLC.

Qreg in Stata https://www.stata.com/manuals14/rqreg.pdf
